# Descending neurons from the lateral accessory lobe and posterior slope in the brain of the silkmoth *Bombyx mori*

**DOI:** 10.1038/s41598-018-27954-5

**Published:** 2018-06-25

**Authors:** Shigehiro Namiki, Satoshi Wada, Ryohei Kanzaki

**Affiliations:** Research Center for Advanced Science and Technology, The University of Tokyo, 4-6-1 Komaba, Meguro, Tokyo 153-8904 Japan

## Abstract

A population of descending neurons connect the brain and thoracic motor center, playing a critical role in controlling behavior. We examined the anatomical organization of descending neurons (DNs) in the brain of the silkmoth *Bombyx mori*. Moth pheromone orientation is a good model to investigate neuronal mechanisms of behavior. Based on mass staining and single-cell staining, we evaluated the anatomical organization of neurite distribution by DNs in the brain. Dense innervation was observed in the posterior–ventral part of the brain called the posterior slope (PS). We analyzed the morphology of DNs innervating the lateral accessory lobe (LAL), which is considered important for moth olfactory behavior. We observed that all LAL DNs also innervate the PS, suggesting the integration of signals from the LAL and PS. We also identified a set of DNs innervating the PS but not the LAL. These DNs were sensitive to the sex pheromone, suggesting a role of the PS in motor control for pheromone processing. Here we discuss the organization of descending pathways for pheromone orientation.

## Introduction

Male moths orient toward conspecific females based on their sex pheromones. Sex pheromones have been demonstrated to reliably elicit stereotyped behavior in moths, and hence are a good model for investigating the general mechanisms underlying olfactory navigation^1^. A specific group of descending neurons (DNs), which connect the brain and ventral nervous system, have been identified as an important element for pheromone orientation in the silkmoth *Bombyx mori*^2^. These DNs have neurites innervating a specific brain region called the lateral accessory lobe (LAL), which is a key area in insect brains connecting the central complex with other parts of the protocerebrum^3,4^. Only a fraction of DNs innervate the LAL and the individual morphology is largely unknown for DNs innervating other brain regions in the moth. Insects have 300–500 DNs from each hemisphere^5–8^. Recent studies have reported that the number of DNs from the LAL is smaller than that from other brain regions in the fruitfly *Drosophila melanogaster*^9^.

The posterior ventral part of the brain can be densely labeled using backfill labeling from the neck connective in insects^5–7,10,11^. A systematic analysis of individual DN morphology revealed that most DNs originate from the area known as the posterior slope (PS) in Drosophila^9^. The PS is densely labeled using backfill staining, suggesting it to be the major origin of DNs in *B. mori*, as in other species^12^. The neuroanatomy of DN innervation in the PS is, however, largely unknown in *B. mori* and its functional role in pheromone orientation remains unclear. Here, we investigated the morphology of individual DNs in the silkmoth using single-cell and backfill labeling techniques. We describe the gross anatomical organization of the descending pathway, focusing on the innervation of the PS.

**Figure 1:**
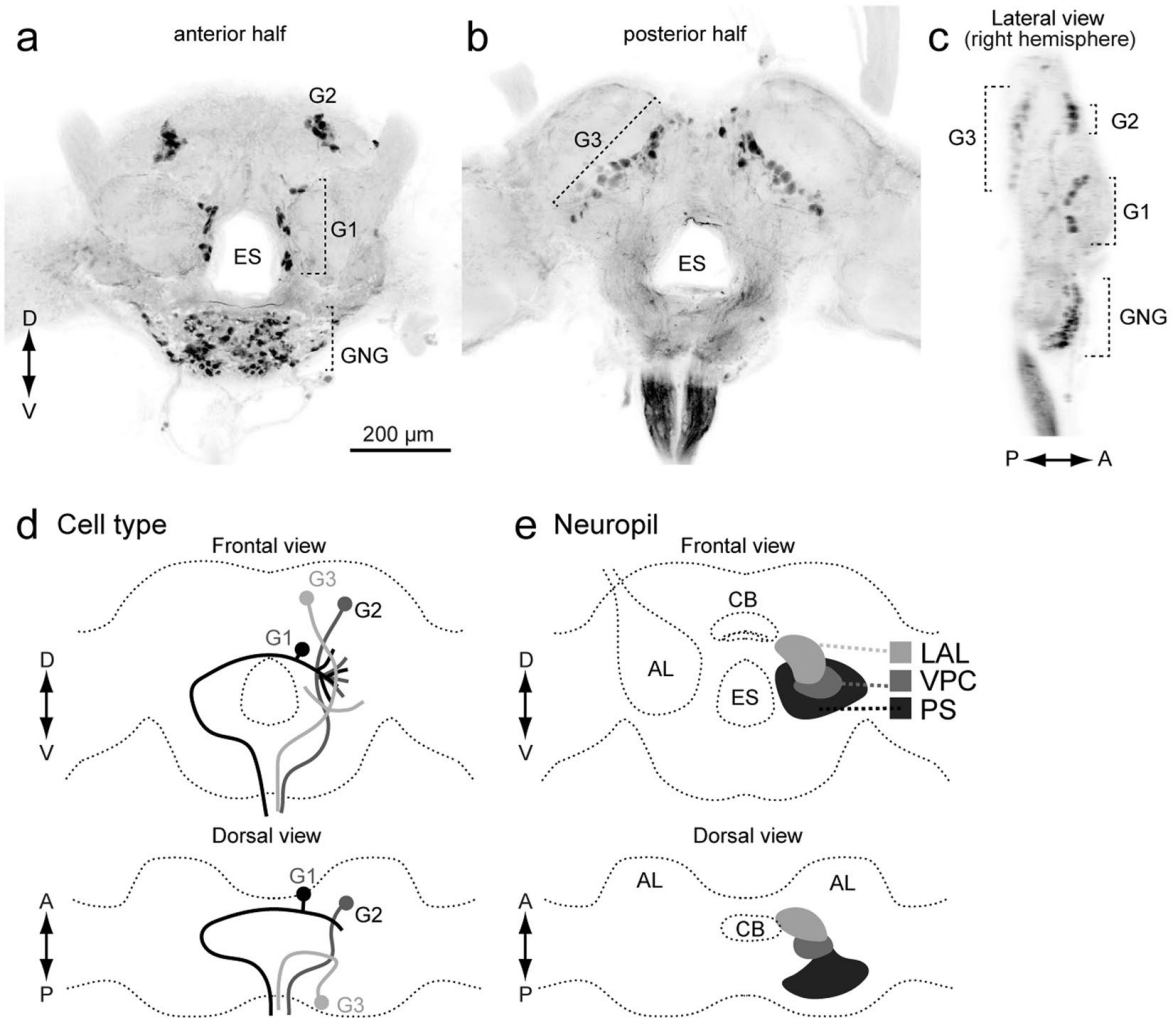
Basic anatomy of descending neurons and major innervation area in the silkmoth brain. (**a**,**b**) Maximum intensity projection of anterior (**a**) and posterior parts of a brain sample stained by the backfilling technique (**b**). (**c**) Lateral view of the brain sample shown in (**a**,**b**). (**d**) Classification of DNs based on the cell body location. (**e**) Schematic of neuropils in the silkmoth brain. Frontal and dorsal views are shown. AL, antennal lobe; CB, central body; ES, esophagus; G1, group-I DNs; G2, group-II DNs; G3, group-III DNs; GNG, gnathal ganglion; LAL, lateral accessory lobe; PS, posterior slope; VPC, ventral protocerebrum.

In a previous study, we classified DNs into three groups based on the cell body location (Fig. 1)^2^. Group-I and -II DNs, whose cell bodies are located on the anterior surface (Fig. 1), and group-III DNs, whose cell bodies are located on the posterior surface, have been identified thus far in *B. mori*^2,13^. The pheromone-evoked flip-flop firing activity, which correlates with the walking direction during pheromone orientation^14^, has been observed in neurons innervating the LAL^12,15^. In the present study, we found that these LAL DNs also have neurite innervation in the PS and in the LAL. In addition, we identified several types of group-III DNs innervating the PS. The organization of the descending pathway for pheromone orientation is discussed based on the neuroanatomy.

## Results

### Organization of descending neurons in the brain

**Figure 2:**
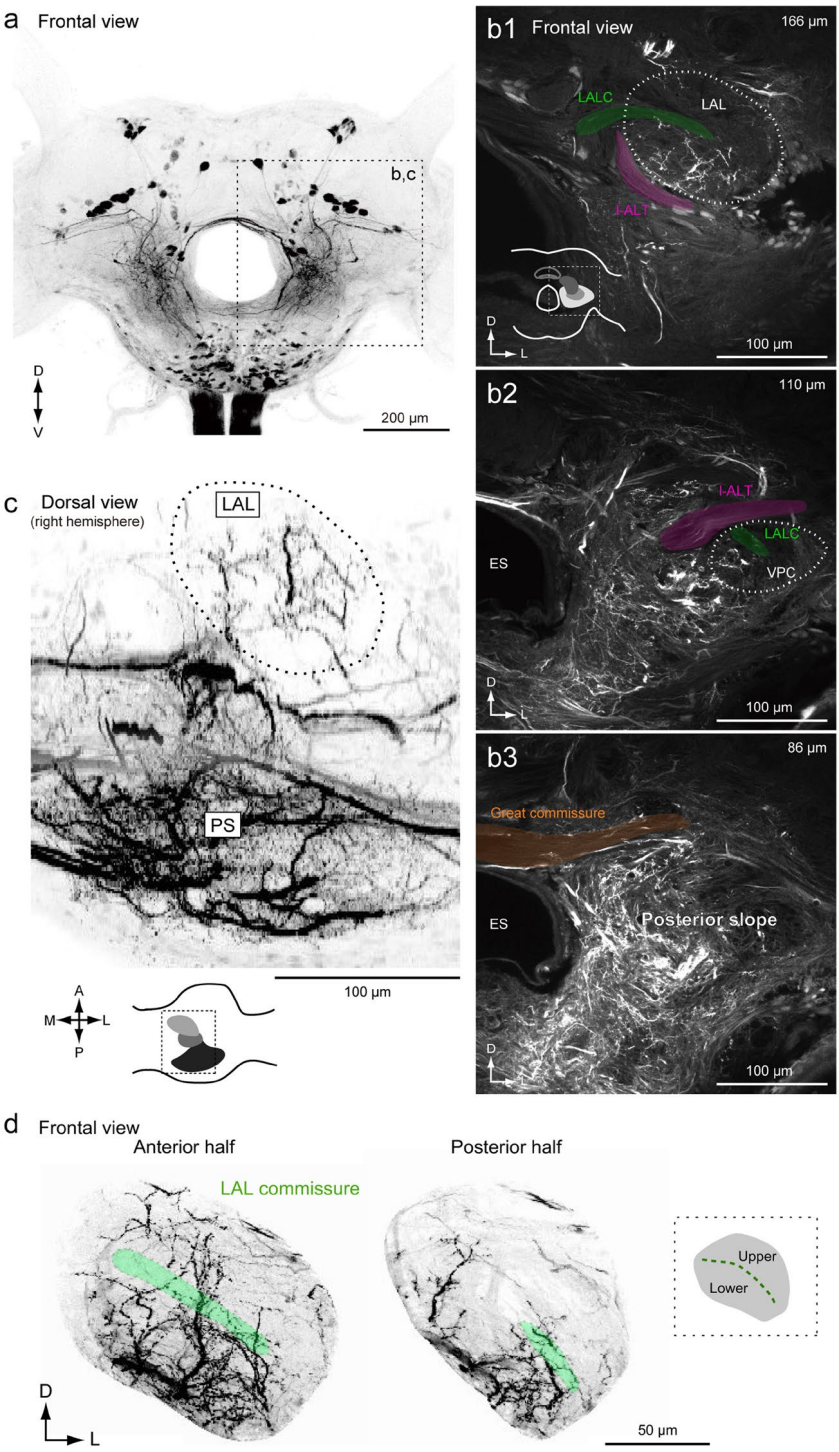
Anatomical organization of descending and ascending neuron innervation in the brain. (**a**) Mass-staining results by backfilling from the neck connective on both sides. The positions of DN groups are shown on the *right*. G1, group-I DNs; G2, group-II DNs; G3, group-III DNs; GNG, DNs of gnathal ganglion. (**b**) Confocal stacks of mass-staining results in the protocerebrum. The depth from the posterior brain surface are shown in the *top-right*. Inset shows the schematic of the imaging area (b1). The LAL and VPC are shown with a broken line. The locations of major bundles are indicated by color: the lateral accessory lobe commissure (LALC, green), lateral antennal-lobe tract (l-ALT, magenta) and great commissure (GC, orange). (**c**) Dorsal view of innervation of descending and ascending neurons. Inset shows the imaging area. The LAL is shown with a broken line. The innervation in the PS is more dense than in the LAL. (**d**) Neurite innervation in the LAL. The maximum intensity projection for the anterior and posterior half of the LAL are shown. The position of the LALC is indicated by green. Inset shows subdivisions within the LAL. The upper and lower divisions are roughly delineated by the LALC. Innervation is observed in both upper and lower divisions in the anterior part, but is largely confined to the lower division in the posterior part of the LAL.

To characterize the anatomical organization of DNs, we performed backfill labeling from the neck connective using florescent dye (Fig. 2). The backfill labeling technique selectively labels DNs and ascending neurons (ANs). A representative example of backfill labeling is shown in Supplementary Video 1. The LAL is located lateral to the central body (Fig. 1e). The LAL has been identified as a brain region with dendritic innervation by DNs, some of which exhibit the characteristic flip-flop response, which is thought to mediate pheromone orientation behavior^2,13^. The ventral protocerebrum (VPC) is well connected with the LAL. There are interneurons whose dendrites are confined within the LAL and VPC, but no interneurons whose dendrites are confined within the VPC have been reported^16^. The PS is an unstructured neuropil located posterior to the VPC (Supplementary Fig. 1). Figure 2b shows confocal images of staining at three different depths corresponding to the LAL, VPC, and PS of the left hemisphere. The posterior part of the protocerebrum, including the PS, was more densely labeled than the anterior part, including the LAL and VPC (Fig. 2c; Supplementary Fig. 2). Furthermore, the posterior lateral protocerebrum (PLP) and ventral lateral protocerebrum (VLP), which mainly receive input from the lobula complex and inferior bridge (IB) located beside the protocerebal bridge, were labeled (Supplementary Fig. 3; Supplementary Video 1). The innervation to the PLP was further extended to the lobula (Supplementary Fig. 3b). The LAL is classified into two subdivisions roughly delineated by the lateral accessory lobe commissure (LALC). Backfilling labeled more innervation in the lower division than in the upper division (Fig. 2d), which is consistent with previous observations based on single cell morphology^16^.

**Figure 3:**
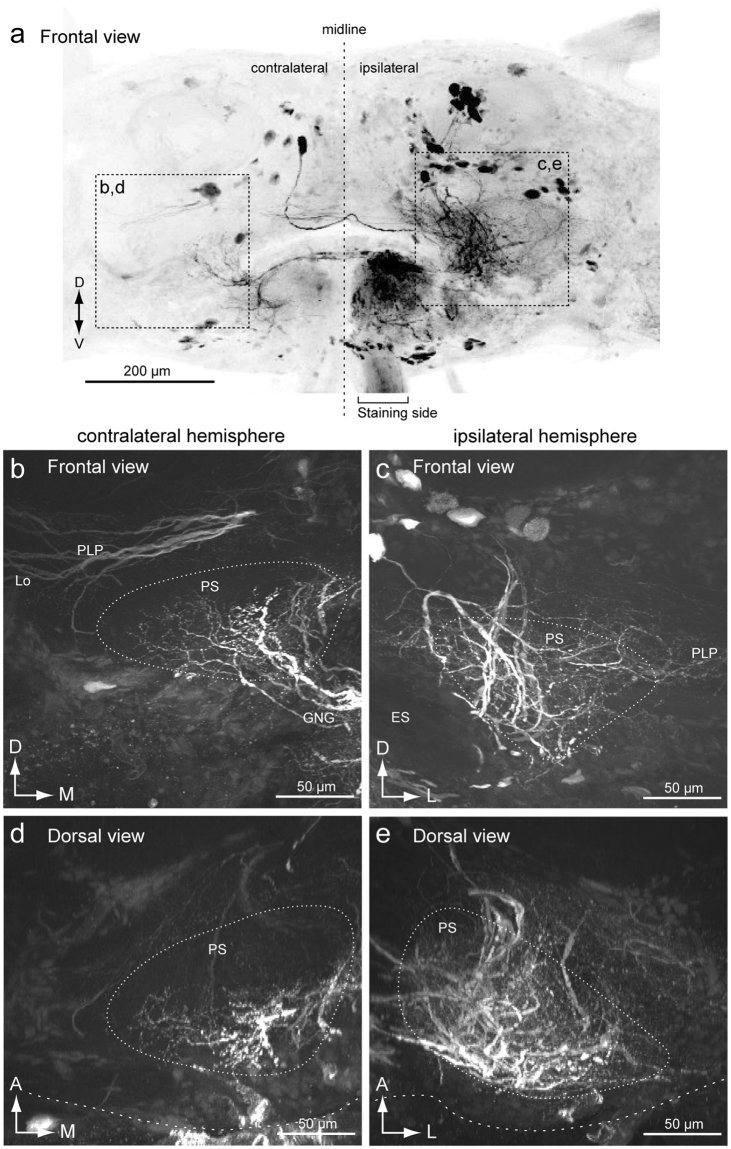
Mass-staining result by backfilling from one side of the neck connective. (**a**) Maximum intensity projection of mass-staining result. The frontal view is shown. The side of filling is noted. (**b**,**c**) Frontal view of the innervation in the posterior slope (PS) of the contralateral (**b**) and ipsilateral sides (**c**). Wide field innervation is observed in the ipsilateral hemisphere. (**d**,**e**) Dorsal view of the innervation in the PS of the contralateral (**d**) and ipsilateral sides (**e**). The innervation is extended more anteriorly in the ipsilateral hemisphere. ES, esophagus; GNG, gnathal ganglia; PLP, posterior lateral protocerebrum.

To compare the neurite innervation in both hemispheres, we examined backfilling from one side of the neck connective (Fig. 3). This resulted in bilateral labeling of the PS, indicating the presence of both ipsilaterally and contralaterally descending fibers from the PS. Innervation to the side of dye injection (ipsilateral side) was broader than those to the contralateral side (Fig. 3b,c). The contralateral innervation was dense in the posterior side and innervation of the anterior side was relatively sparse (Fig. 3d). Biased innervation within the LAL (Fig. 2d) was also confirmed when we filled one side of the neck connective (Supplementary Fig. 4).

Furthermore, to characterize the anatomical organization at single-cell resolution, we analyzed the morphology of individual DNs (Supplementary Table 1). The total number of DNs in the insect brain is estimated to be 200–500^5–7^. We analyzed a total of 57 DNs (at least 24 different cell types) in the present study. Among them, 38 and 19 DNs descended the neck connective that arises from the ipsilateral and contralateral hemispheres to the cell body, respectively. Twenty-two DNs had branches crossing the midline. Hereafter we use the term ‘ipsilateral’ for the side where the cell body is present, and ‘contralateral’ for the opposite side.

We focused on the terminal morphology of neurons in particular. Because the morphological features are correlated with pre- and postsynaptic structures, and are therefore a good indicator for polarity of neurons^17^. An electronmicroscopic study systematically quantified synapse volume in Drosophila, and reported that postsynaptic profiles were small in diameter and presynaptic profiles were large in diameter^17^ and similar observations have been reported in other insects^18–21^. The terminal morphology of the neuron in *B. mori* was either smooth or varicose in appearance (Supplementary Fig. 5). Smooth processes usually had neurites with a small diameter and often exhibited dense arborization. Varicose processes had neurites with a large diameter and their distribution was sparse. Smooth processes were usually located more closely to the cell body position than varicose processes. In the case of bilateral neurons which connected both hemispheres, the neurite in the hemisphere containing the cell body (ipsilateral) had a smooth appearance, whereas the neurite in the contralateral hemisphere had a varicose appearance (Supplementary Fig. 5c,d).

Most DNs had varicose processes in the gnathal ganglion (GNG) (98%, *n* = 57) and 22 DNs had varicose processes in the PS (39%, *n* = 57). The PS was the region that contained the largest number of DNs in the brain (86%, *n* = 57). Almost all DNs suppled varicose processes in the GNG in the silkmoth (Supplementary Table 1), suggesting that most DNs provide output signals to both the ventral nervous system and GNG. This anatomical feature is also observed in Drosophila, where 78% of DN types have output terminals in the GNG^9,22^.

### Group-I descending neurons

**Figure 4:**
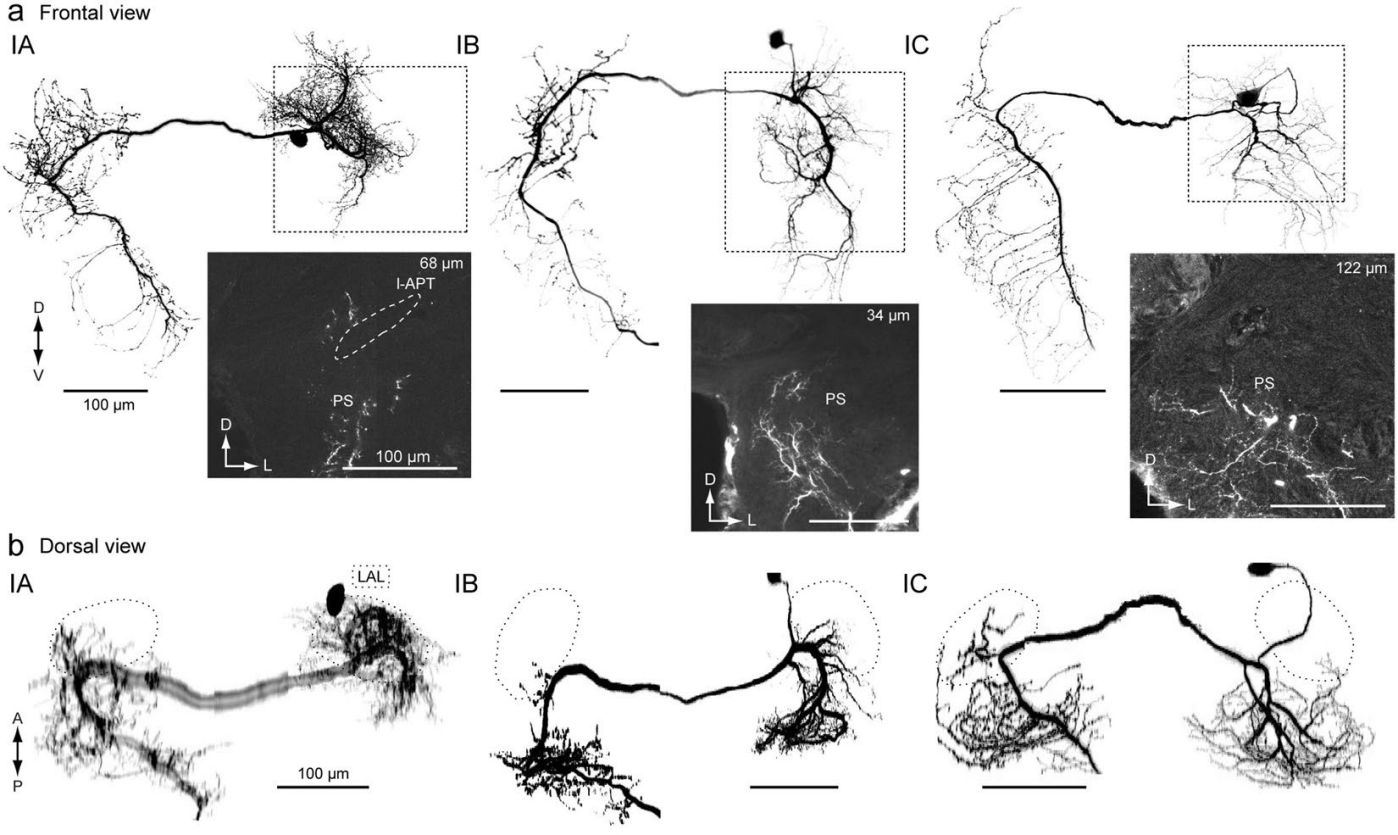
Neurite innervation to the posterior slope PS by group-I and group-II descending neurons. (**a**) Morphology of group-I DNs. Maximum intensity projection of all innervation in the brain (*top*) and confocal stack of the neurite innervation in the posterior slope (PS) are shown (*bottom*). Frontal view is shown. Group-IA, group-IB and group-IC innervate a part of the medial PS, medial PS and entire PS, respectively. The original data are taken from^13^ for the group-IB DN. (**b**) Dorsal view of DN morphology. The lateral accessory lobe (LAL) is shown with a broken line. There is dense innervation outside the LAL. A major neurite of the group-IA DN exhibited halation due to strong illumination during imaging. l-APT, lateral antennal-lobe tract.

We analyzed a total of 11 group-I DNs (Supplementary Table 1). Figure 4 shows the morphology of three types of group-I DNs: group-IA, group-IB and group-IC DNs^2,13^. We reexamined the morphology focusing on the innervation outside the LAL. They are contralaterally descending (91%, n = 11). Group-I DNs have smooth processes in the LAL and PS of the ipsilateral hemisphere, and varicose processes in the PS and GNG of the contralateral hemisphere. Group-IA DN has smooth processes in the small portion of the medial PS of the ipsilateral hemisphere and varicose processes in anterior medial part of the PS of the contralateral hemisphere (Fig. 4; Supplementary Fig. 6). Group-IB DN has smooth processes in the medial PS of the ipsilateral hemisphere covering a wider area than group-IA (Fig. 4; Supplementary Fig. 7). Group-IB has varicose processes in the medial PS of the contralateral hemisphere, which is different from group-IA. In contrast, the smooth processes of group-IC DN occupy the entire volume of the PS of the ipsilateral hemisphere (both medial and lateral PS), but varicose processes occupy the PS of the contralateral hemisphere, which is similar to those of Group-IB (Fig. 4; Supplementary Fig. 8). Smooth innervation differs in arborization and the varicose processes exhibit biased innervation toward the medial PS.

**Figure 5:**
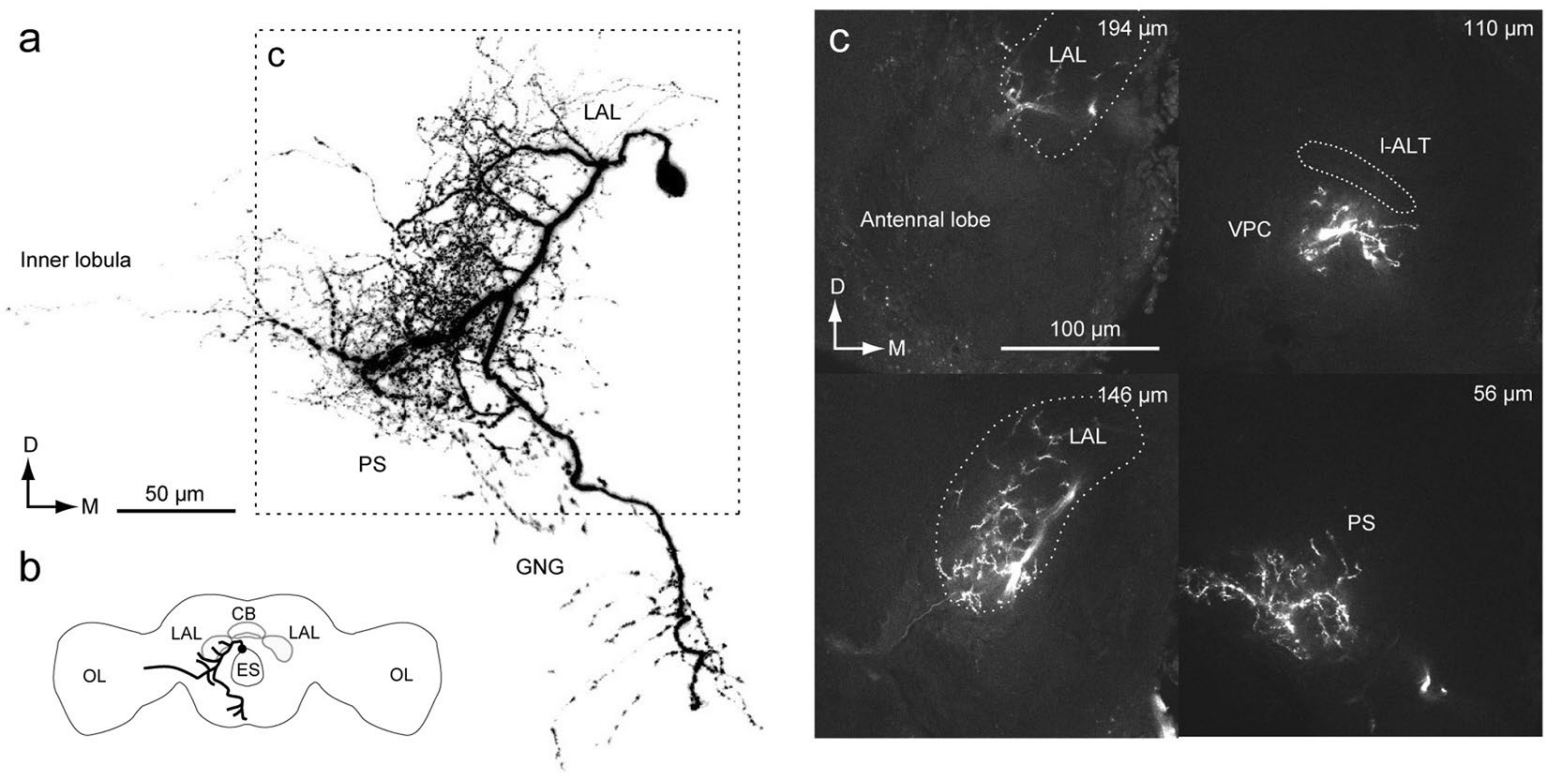
Newly identified group-ID DN innervating the LAL. (**a**) Whole morphology of the DN. The DN has smooth processes in the ipsilateral lateral accessory lobe (LAL), ventral protocerebrum (VPC) and posterior slope (PS), and varicose processes in the ventral PS and GNG. The axon runs through the medial route in the GNG and descends the ipsilateral neck connective. (**b**) Schematic of DN morphology. (**c**) Confocal stacks of neurite innervation. The depth from the posterior brain surface is shown in the *top-right*. The innervation is biased toward the lateral side within the LAL. The innervation in the PS is biased toward the lateral side.

**Figure 6:**
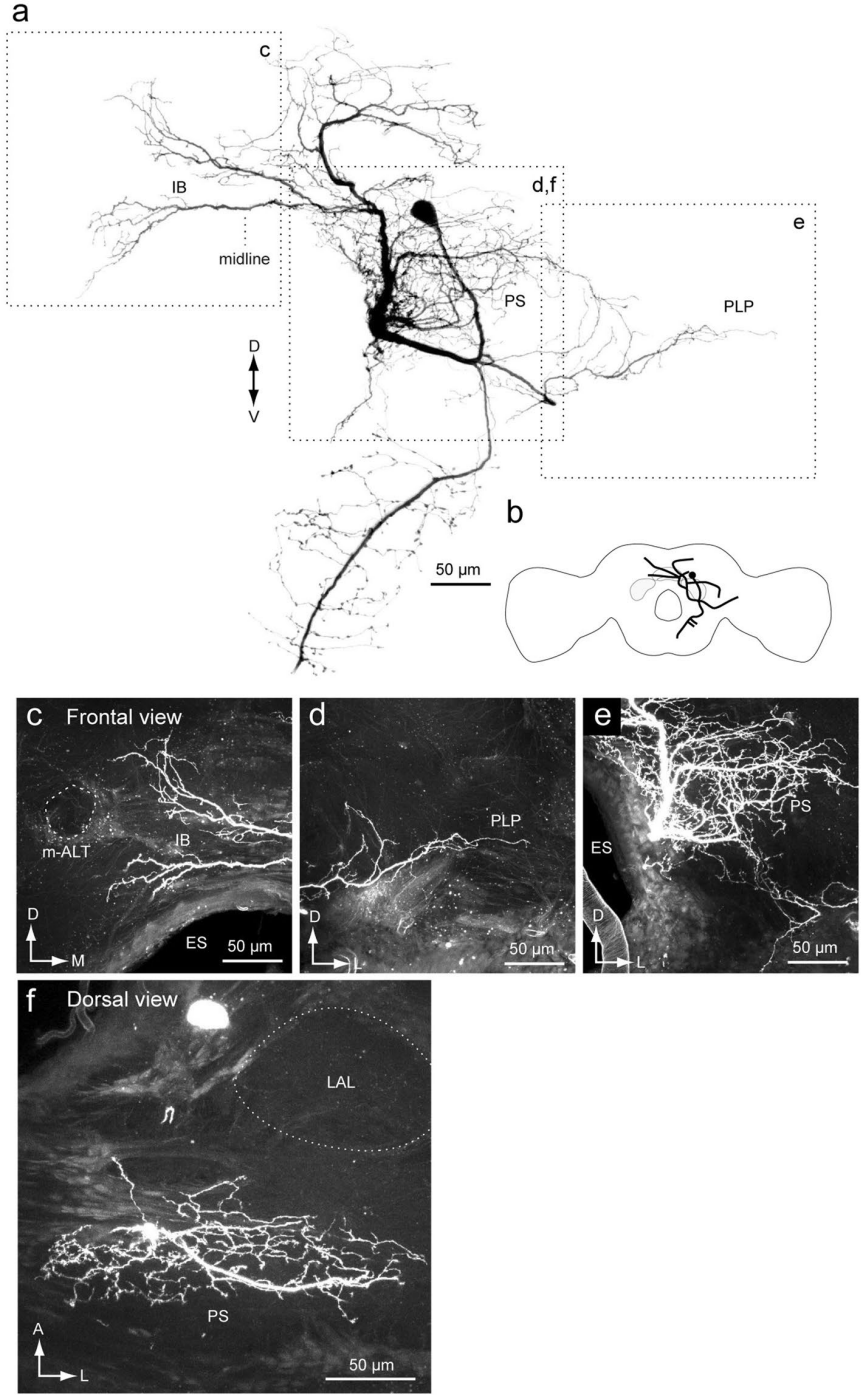
Newly identified group-IE DN. (**a**) Whole morphology of the DN. (**b**) Schematic of the neuronal morphology. DN has smooth processes in the inferior bridge (IB) (**c**), posterior lateral protocerebrum (PLP) (**e**) and the posterior slope (PS) (**e**). Unlike other group-I DNs, the DN does not have innervation to the lateral accessory lobe (LAL) (**f**). ES, esophagus; m-ALT, medial antennal-lobe tract.

In addition to the types described thus far, we identified novel types of group-I DNs, whose cell bodies are located on the anterior brain surface (Figs 5 and 6). Figure 5 shows the morphology of a unilateral DN, referred to as group-ID. The DN has smooth processes in the LAL, VPC and PS, and varicose processes in the PS and GNG of the ipsilateral hemisphere. Thin neurites extends to the PLP and inner lobula (Fig. 5a). The innervation within the LAL is biased toward the lower division (Fig. 5c), similar with the other types of group-I DNs. The innervation in the PS is biased toward the lateral side. Unlike other group-I DNs, this DN descends to the ipsilateral neck connective.

Figure 6 shows another example of group-I DNs, referred to as group-IE. The DN has smooth processes covering a wide area in the protocerebrum, including the PS and IB, and varicose processes in the GNG. Some neurites in the inferior bridge crosses the midline. The lateral branch reaches the PLP. Unlike the other group-I DNs, the group-IE DN does not innervate the LAL.

### Group-II descending neurons

**Figure 7:**
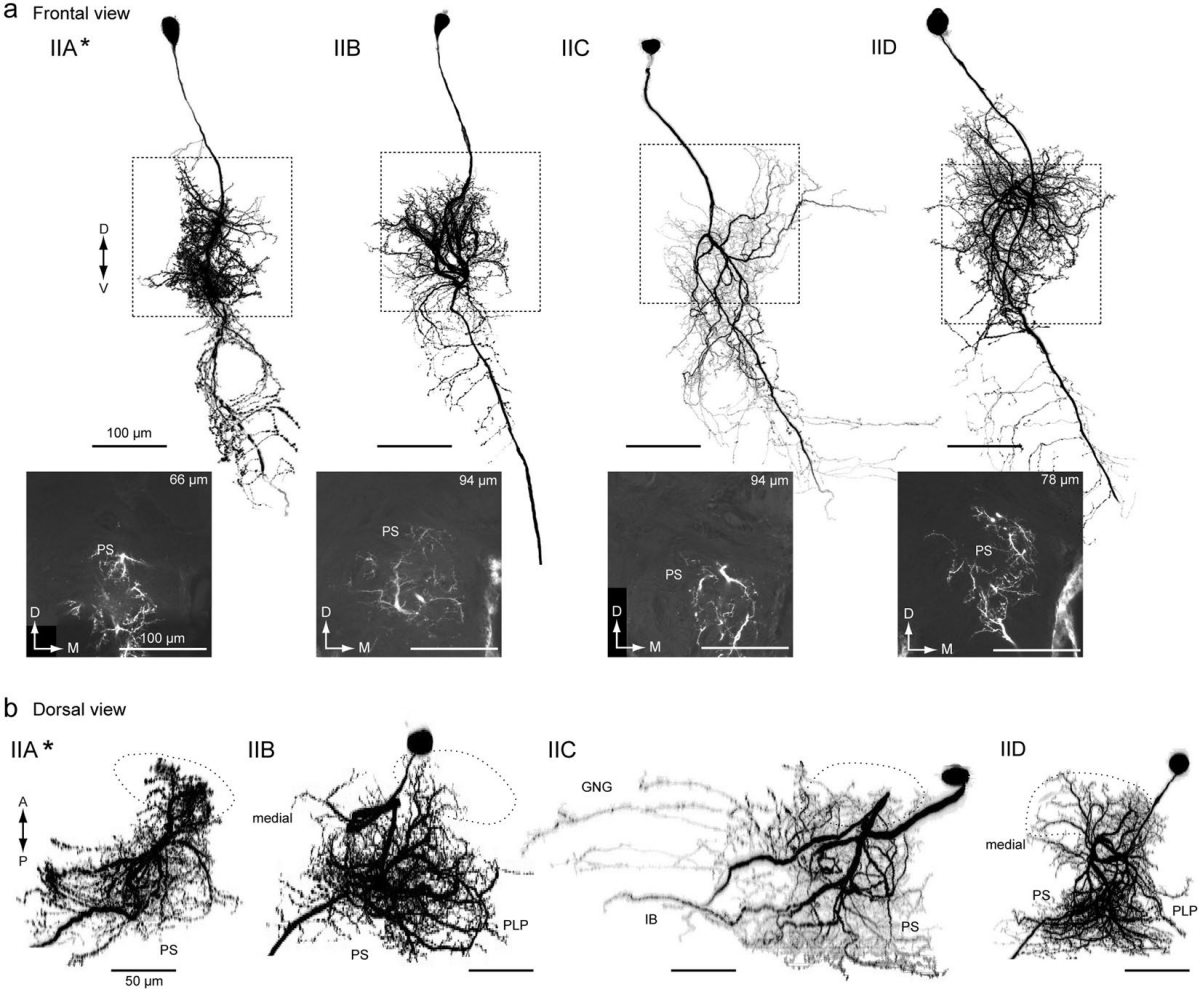
Group-II DNs innervate the PS. (**a**) Morphology of group-II DNs. Maximum intensity projection of all innervation in the brain (*top*) and confocal stack of the neurite innervation in the posterior slope (PS) are shown (*bottom*). The frontal view is shown. A flipped image is shown for comparison (asterisk). All DN types have innervation in the medial PS. (**b**) Drosal view of DN innervation in the brain. The LAL is shown with a broken line. The innervated areas are similar among group-II DNs, in comparison with group-I DNs (Fig. 4). GNG, gnathal ganglion; IB, inferior bridge; PLP, posterior lateral protocerebrum; PS, posterior slope.

We analyzed 29 group-II DNs, which are classified into a total of six cell types (group-IIA–F, Supplementary Table 1). Four types have been identified thus far^2,13^, and two types were newly identified in the present study. All types are ipsilaterally descending, and have smooth processes in the PS and varicose processes in the GNG of the ipsilateral hemisphere. Figure 7 shows the morphology of group-II DNs. The group-IIA DN innervates the LAL, VPC and medial part of the PS, which is located medial to the LALC. Moreover, the DN has a number of branches entering the unstructured region medial to the LAL. These branches run dorsal and ventral to the lateral antennal lobe tract (l-ALT) (Supplementary Fig. 9a, asterisks). In the deep PS, the innervation is biased toward the dorsal side. The ventral branch is bifurcated and enters the GNG. The group-IIA DN also has branches in the ventral end of the superior medial protocerebrum (SMP).

The group-IIB DN innervates the LAL, VPC and medial part of the PS (Supplementary Fig. 9b). As in group-IIA, the group-IIB DN has branches in the ventral end of the SMP. The volume of innervation in the PS is wider than for the other types of group-II DNs. The DN has smooth branches located posterior to the LAL and dorsal to the l-ALT. The axon runs through the medial route of the GNG with side branches.

The group-IIC DN innervates the VPC and medial side of the PS. The DN has additional processes in the inferior bridge (Fig. 7, Supplementary Fig. 9c), which is posterior of the midline region behind the central body upper division and below the protocerebral bridge^23–25^. The medial branch into the inferior bridge often crosses the midline. The axon runs through the medial route in the GNG and side branches extend laterally; there are fewer side branches than in the other types.

The group-IID DN innervates the LAL, VPC and medial side of the PS (Supplementary Fig. 9d). The innervation in the LAL is mostly localized to its lower division and the anterior portion of the upper division. Some processes reach the ventral end of the SMP, and some branches invaded the periesophageal area located medial to the LAL; the axons ran through the medial route in the GNG with many side branches toward the lateral side.

**Figure 8:**
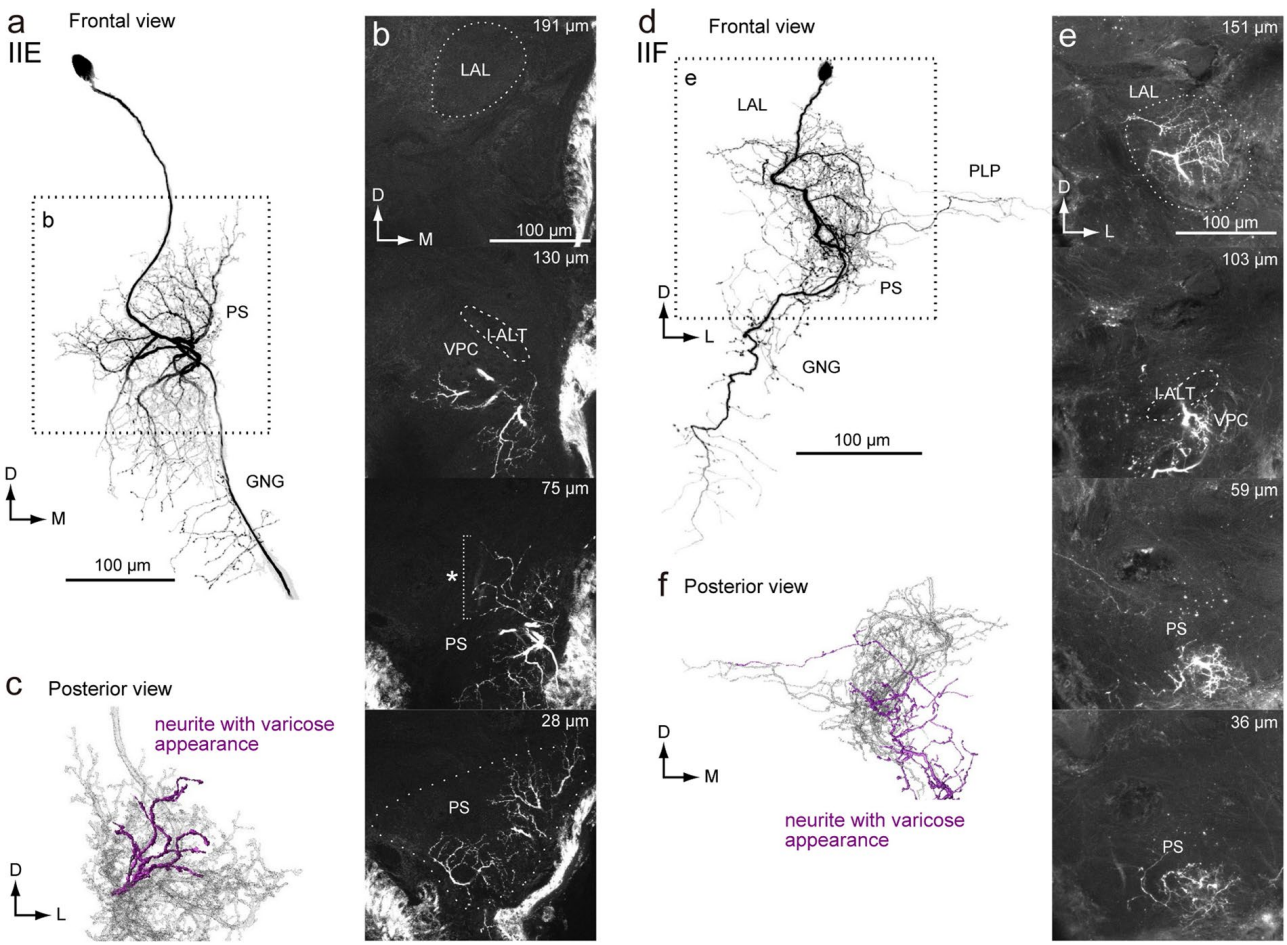
Newly identified group-II DNs. (**a**) Maximum intensity projection of the DN innervation in the brain. The morphology is similar with the group-IID DN. Unlike to other group-II DNs, the DN has innervation with varicose appearance in the PS. The DN also innervates the PLP. (**b**) Confocal stacks for the DNs in (**a**). The DN has innervation in the almost all of the LAL, the ventral protocerebrum (VPC) and the medial part of the inferior PS. The depth from the posterior surface is shown in the *top-right*. (**c**) Posterior view of three-dimensional reconstruction of the DN in the PS. The neurites with varicose appearance are shown in magenta. (**d**) Maximum intensity projection of the DN innervation in the brain. The morphology is with the group-IIC DN. Unlike other group-II DNs, the DN has innervation with varicose appearance in the PS. (**e**) Confocal stacks for the DNs in (**a**). The DN has innervation in the almost all of the LAL, the VPC and the inferior PS. The depth from the posterior surface is shown in the *top-right*. (**f**) Posterior view of three-dimensional reconstruction of the DN in the PS. The neurites with varicose appearance are shown in magenta.

In addition, we identified two novel types of group-II DNs (Fig. 8). The DN innervates the PS, but not the LAL or VPC. Although its morphology is similar with the group-IIC DN, some of the innervation supplies to the PS have a varicose appearance (Fig. 8c), which is likely presynaptic^17^. The DN is referred to as the group-IIE DN. Another example, the group-IIF DN is similar in morphology with the group-IID DN (Fig. 8d). The DN innervates the entire area within the LAL, VPC and medial part of the PS. The innervation in the PS is mostly smooth in appearance but also has some varicose areas (Fig. 8f). A few branches extend to the PLP. Overall, all group-II DNs have smooth processes in the medial side of the PS, suggesting a shared input region. All three types of group-I DNs have varicose processes in the medial PS of the contralateral hemisphere, overlapping with the smooth processes of group-II DNs, suggesting interaction between group-I and group-II DNs.

### Group-III descending neurons

We identified a total of 17 group-III DNs (Supplementary Table 1). Areas highly innervated by DNs were the PLP (10 DNs), PS (9), lobula (5), SMP (5), inferior bridge (4), and inferior clamp (4) (Supplementary Figs 10 and 11). Ten DNs descended contralaterally, and seven DNs descended ipsilaterally. As in group-I and group-II DNs, most group-III DNs had varicose processes in the GNG (94%, *n* = 17) and nine out of 17 DNs had varicose processes in the PS (53%).

**Figure 9:**
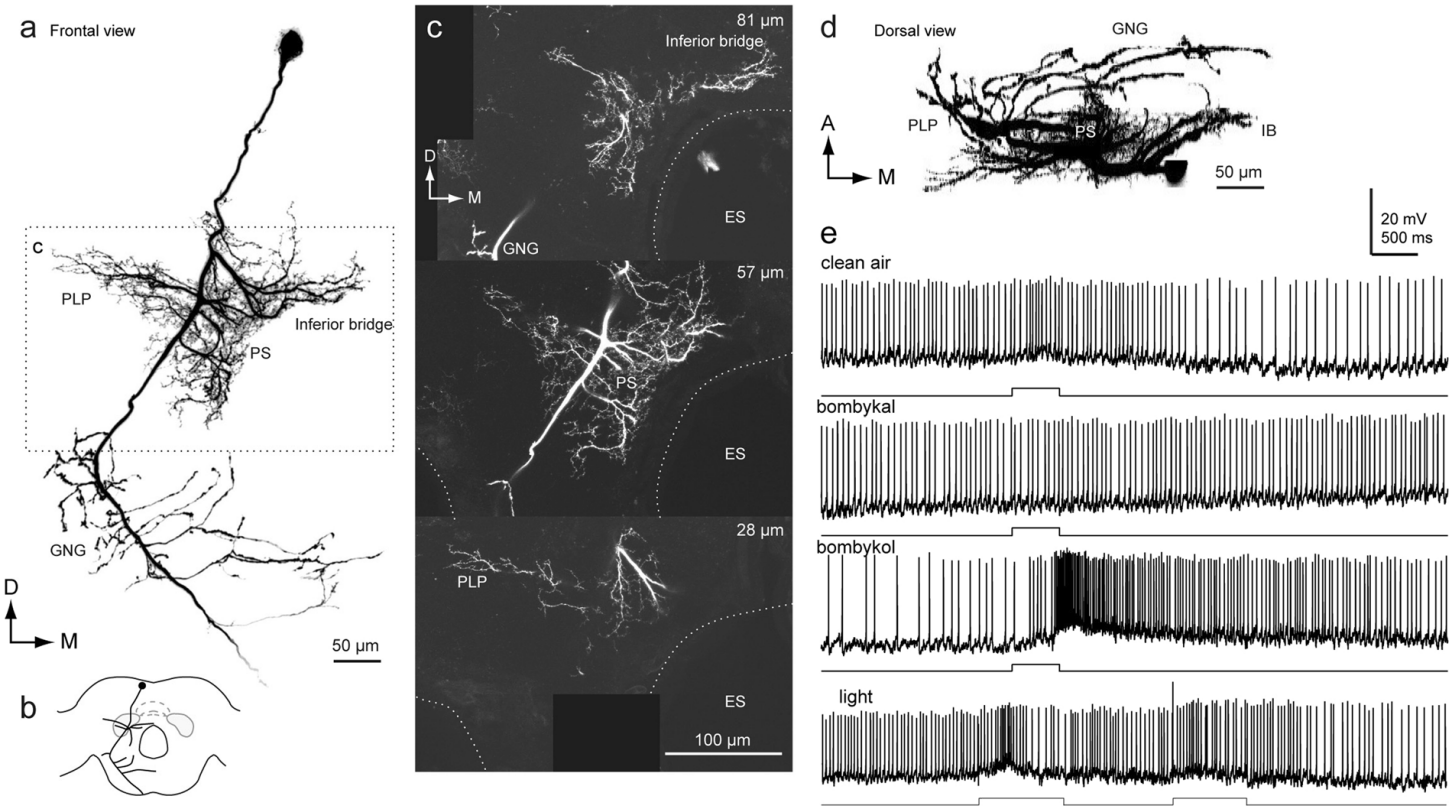
Morphology of a group-III DN innervating the PS (Group-III PSDN1). (**a**) Maximum intensity projection of the DN innervation in the brain. The neuron has smooth processes in the inferior bridge (IB), posterior lateral protocerebrum (PLP) and posterior slope (PS), and varicose processes in the gnathal ganglion (GNG). (**b**) Schematic of neurite innervation for the neuron shown in (**a**). (**c**) Confocal stacks for the DN shown in (**a**). The depth from the posterior brain surface is shown in the *top-right*. (**d**) Dorsal view of the DN morphology. (**e**) Response properties of the neuron. The neuron exhibited excitatory responses to exposure to bombykol, the sex pheromone. ES, esophagus.

**Figure 10:**
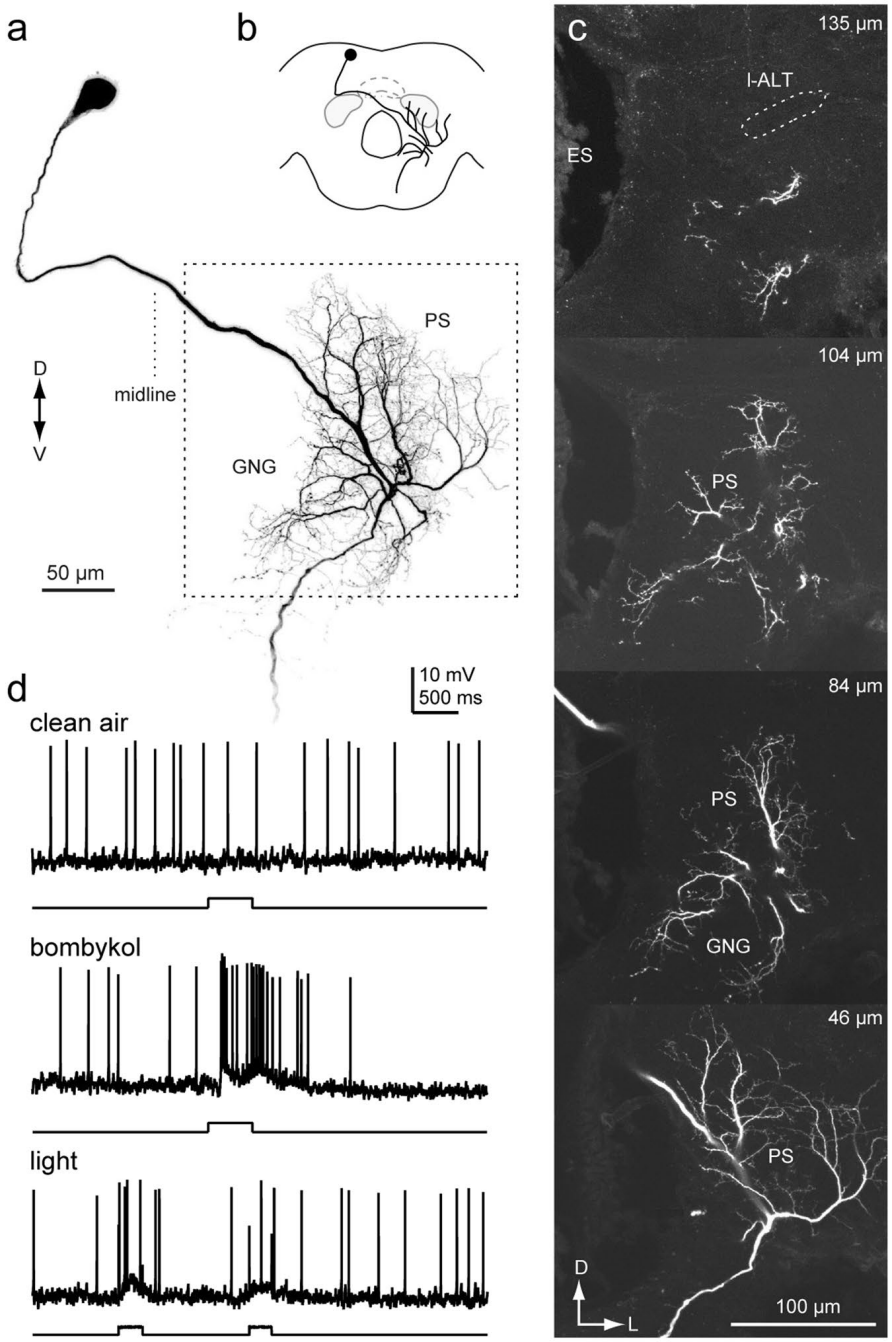
Morphology of a descending neuron innervating the PS (Group-III PSDN2). (**a**) Maximum intensity projection of the DN innervation in the brain. The neuron has smooth processes in the posterior slope (PS) and gnathal ganglion (GNG), and varicose processes in the GNG. (**b**) Schematic of neurite innervation for the neuron shown in (**a**). (**c**) Confocal stacks for the DNs shown in (**a**). The depth from the posterior brain surface is shown in the *top-right*. The neuron lacks innervation in the LAL. ES, esophagus; l-ALT, lateral antennal-lobe tract.

Among these, four DNs innervated the PS but not the LAL (Figs 9 and 10; Supplementary Figs 12 and 13). Figure 9 shows an example of a DN innervating the PS, but not the LAL (group-III PSDN1). The DN has smooth processes in the PS, PLP and IB, and varicose processes in the GNG. The axon runs through the lateral route of the GNG. The DN exhibited an excitatory response to bombykol, the major sex pheromone of *B. mori*, but not to bombykal, a behavioral antagonist^26^. Supplementary Fig. 12 shows another example for group-III PSDN1, which also responded to bombykol.

Figure 10 shows another DN innervating the PS and GNG (group-III PSDN2). The DN has smooth processes in the PS. The processes in the GNG mostly exhibit a smooth appearance, but some neurites have a varicose appearance. The DN dose not innervate known pheromone processing circuits, including the LAL^16^, suggesting that the PS may also process sex pheromone information. In addition, these DNs demonstrated weak excitation in response to light stimulation (Figs 9f and 10d), suggesting the integration of visual and pheromone information in the PS. Supplementary Fig. 13 shows a neuron with the same morphology. Additionally, we identified a neuron connecting the PS and LAL (Supplementary Fig. 14). The neuron exhibited an excitatory response to the sex pheromone and had smooth process in the PS, but not in the LAL. Together with the DNs mentioned above, our results suggest that the PS processes sex pheromone information.

**Figure 11:**
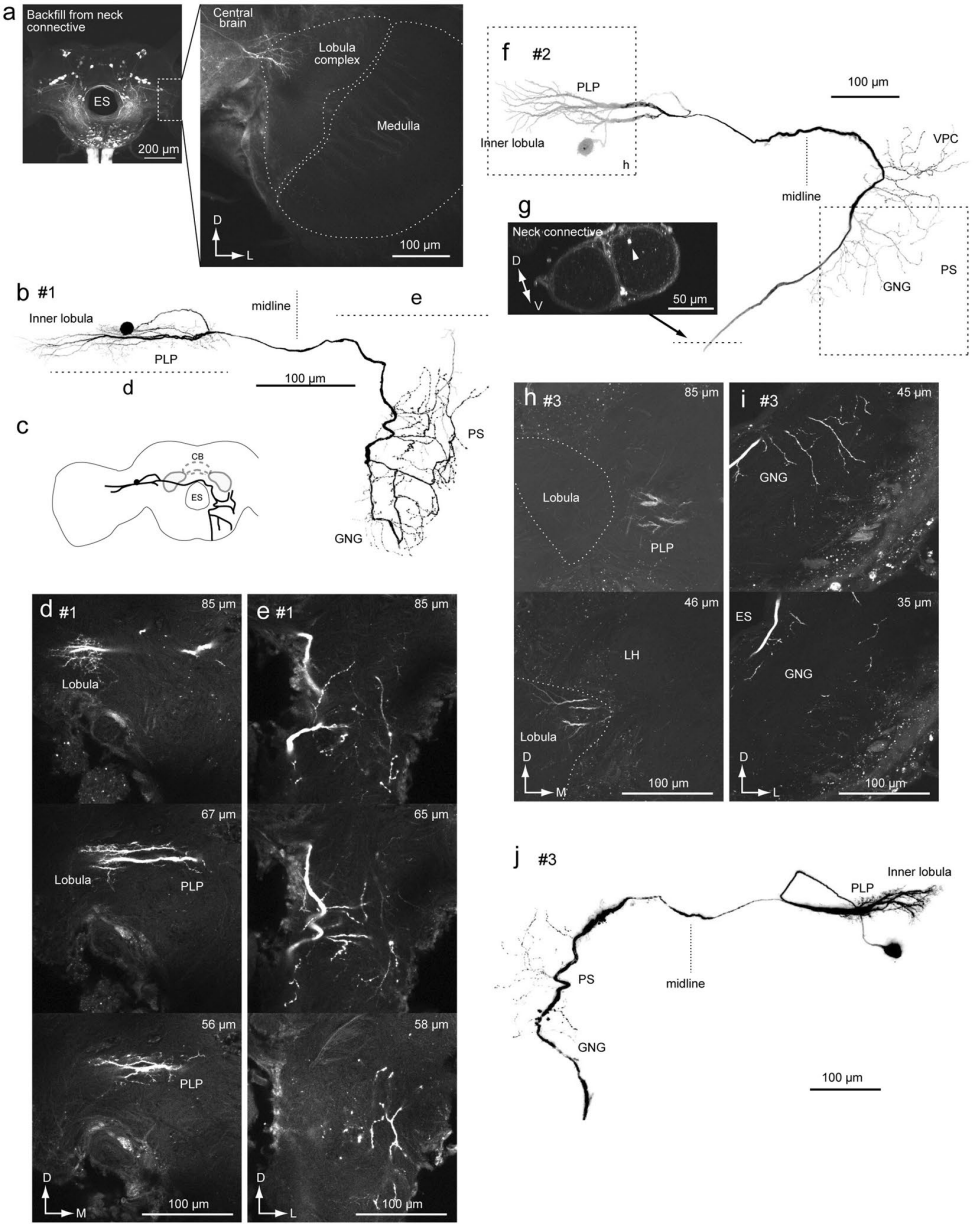
Group-III lobula descending neuron 1 (LDN1). (**a**) An example of backfilling that labeled neurites entering the optic lobe. Whole brain image (*left*) and high magnification image of the optic lobe are shown (*right*). (**b**) Morphology of a DN innervating the inner lobula. The DN has smooth processes in the inner lobula and posterior lateral protocerebrum (PLP) of the ipsilateral hemisphere, and varicose processes in the posterior slope (PS) and gnathal ganglion (GNG) of the contralateral hemisphere. (**c**) Schematic of the neuronal morphology. (**d**,**e**) Confocal stacks of neuronal innervation in the lobula (**d**) and PS (**e**). The depth from posterior brain surface is shown in the *top-right*. (**f**) Another morphology example of group-III LDN1. The basic anatomical features are the same as the DN shown in (**a**). The position in the neck connective (**g**), innervation of smooth processes (**h**) and varicose processes are shown (**i**). (**j**) Third example of group-III LDN1 morphology.

**Figure 12:**
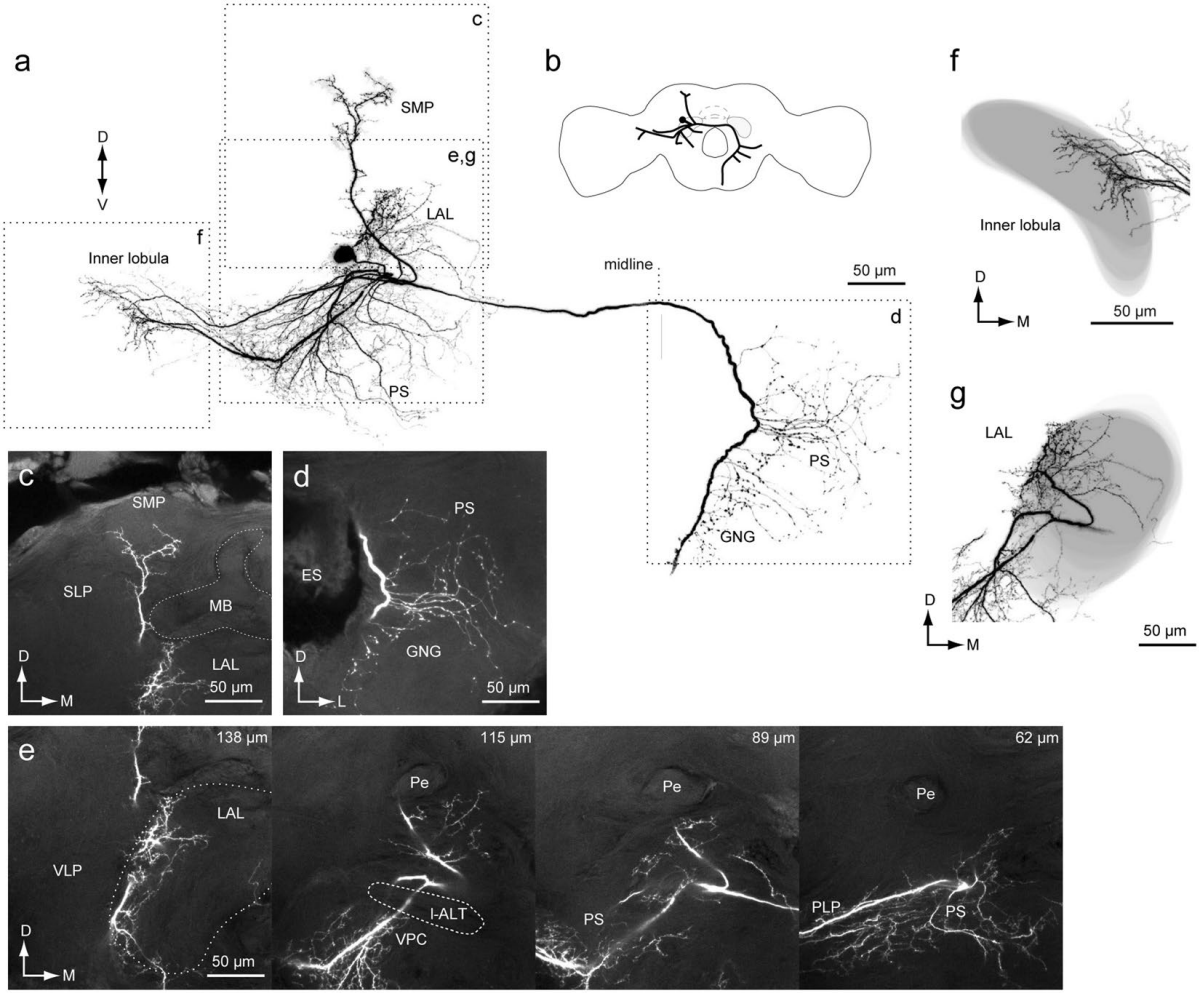
Group-III lobula descending neuron 2: DN with wide field innervation in the brain. (**a**) Neuronal morphology in the brain. The neuron has smooth processes in the inner lobula, posterior lateral protocerebrum (PLP), posterior slope (PS), superior medial protocerebrum (SMP), ventral lateral protocerebrum (VPC) and the lateral accessory lobe (LAL) of the ipsilateral hemisphere, and varicose processes in the PS and gnathal ganglion (GNG) of the contralateral hemisphere. (**b**) Schematic of the neuronal morphology. (**c**–**e**) Confocal stacks for innervation in the SMP (**c**), PS (**d**) and LAL (**e**). The depth from the posterior brain surface is shown in the *top-right* (**e**). (**f,g**) Reconstruction of the neuropil shape and neuronal innervation in the inner lobula (**f**) and LAL (**g**). The innervation is biased toward the medial side in the inner lobula (**f**) and biased toward the lateral side in the LAL (**g**).

**Figure 13:**
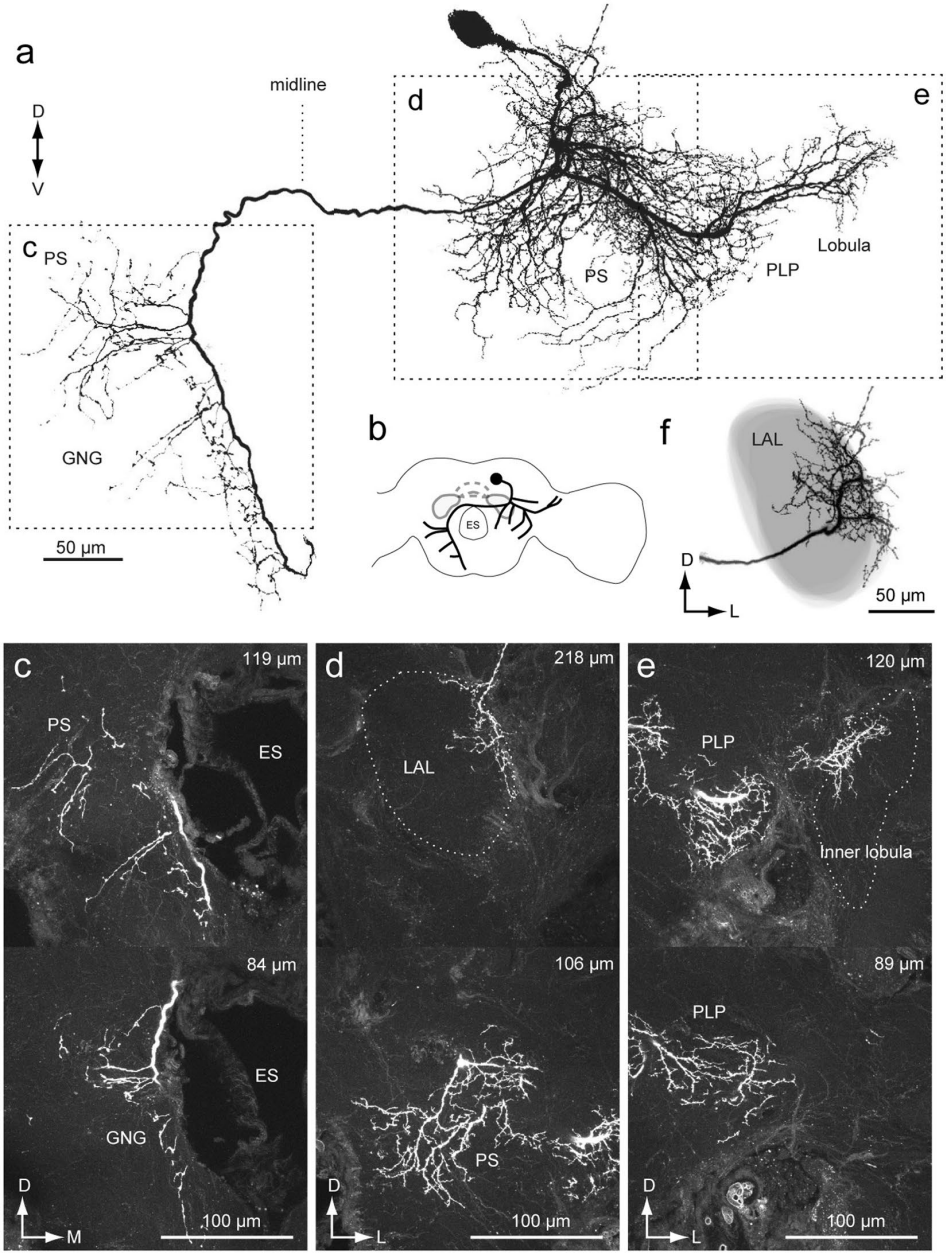
Group-III lobula descending neuron 3: DN with wide field innervation in the brain. (**a**) Neuronal morphology in the brain. The neuron has smooth processes in the inner lobula, posterior lateral protocerebrum (PLP), superior medial protocerebrum, posterior slope (PS), ventral lateral protocerebrum (VPC) and the lateral accessory lobe (LAL) of the ipsilateral hemisphere, and varicose processes in the PS and gnathal ganglion (GNG) of the contralateral hemisphere. The morphology is similar to the neuron shown in Fig. 14. (**b**) Schematic of the neuronal morphology. (**c**–**e**) Confocal stacks for innervation in the GNG (c), LAL and PS (**d**), and PLP and inner lobua (**e**). The depth from the posterior brain surface is shown in the *top-right*. (**f**) Reconstruction of the LAL shape and neuronal innervation. The innervation is biased toward the lateral side of the LAL.

We also identified DNs directly contacting the optic lobe (Figs 11–13). All of these DNs innervated the inner lobula. We did not find DNs innervating the outer lobula or other parts of the optic lobe. Figure 11 presents the morphology of a DN, which has smooth processes in the inner lobula and PLP, but not in the PS. The axon travels to the contralateral hemisphere via the LAL commissure, and supplies varicose processes in the PS and GNG. We refer to this DN as group-III lobula descending neuron 1 (group-III LDN1). We were successful in staining two more DNs with similar morphology as the neuron shown in Fig. 11b.

Figures 12 and 13 provide other examples of DNs innervating the lobula. The DNs have smooth processes in the inner lobula, LAL, PS, and SMP of the ipsilateral hemisphere, and varicose processes in the PS and GNG of the contralateral hemisphere. These DNs have a similar innervation profile, but the shape of innervation differs. We refer to these DNs as group-III lobula descending neuron 2/3 (Group-III LDN2/3).

### DN Innervation in the LAL

**Figure 14:**
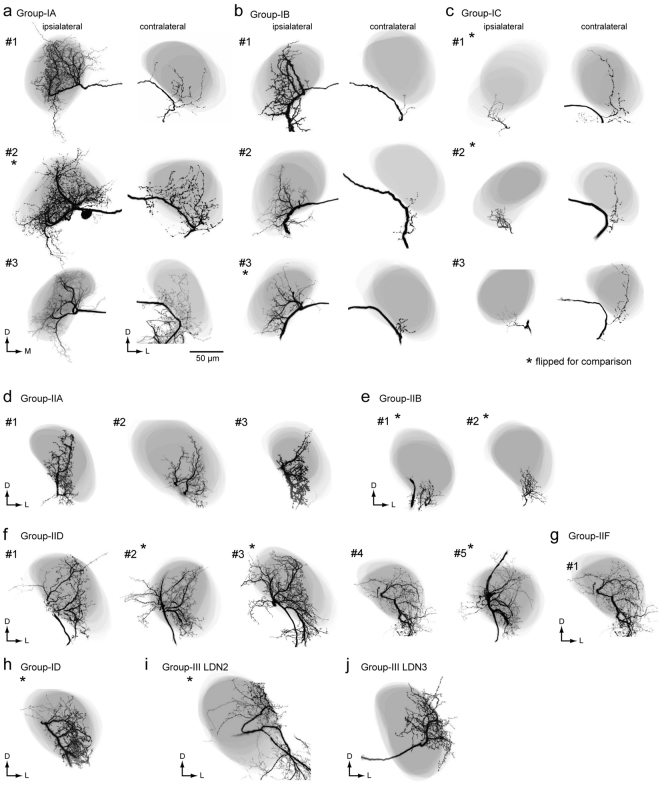
Innervation of the LAL by DNs. Neuronal innervation (black) and the LAL are shown (gray) for group-IA (**a**), group-IB (**b**), group-IC (**c**), group-IIA (**d**), group-IIB (**e**), group-IID (**f**), group-IIF DNs (**g**), group-ID DN (**h**), group-III LDN2 (**i**) and group-III LDN3 (**j**). In some cases, flipped images are shown for comparison (asterisk). Group-IIC and IIE DNs do not innervate the LAL.

In previous studies^2,27^, only four DN types, group-IA, IB, IIA, and IID, were identified as innervating the LAL. In the present study, we identified four novel DN types which innervate the LAL (group-ID, group-IIF, group-III LDN2, and group-III LDN3; Figs 5, 7, 12 and 13). We analyzed the detailed morphology of neurite innervation by reconstructing the LAL volume (Fig. 14). The operational definition for the anatomical boundary of the LAL is defined by Iwano *et al*.^27^. Using this definition, we re-examined their morphology. In the present study, we identified innervation of a small volume within the LAL also by group-IC and group-IIB DNs (Fig. 7). Regarding the anatomy of these DNs, we found that the smooth processes were located above the depth of the saddle point of the l-ALT, which is classified as a part of the LAL^27^. The newly identified group-IIF seemed to innervate the entire LAL, similar with the innervation pattern for group-IID (Fig. 7). We did not find innervation in the LAL by group-IIC or group-IIE DNs. DNs innervating the LAL exhibit biased innervation toward the medial side in most cases (lower division; Fig. 14a–h)^16^. Unlike the other types, group-III LDN2 and LDN3 innervation is biased toward the lateral side of the LAL (upper division; Fig. 14i).

## Discussion

### DN innervation to the LAL and PS

**Figure 15:**
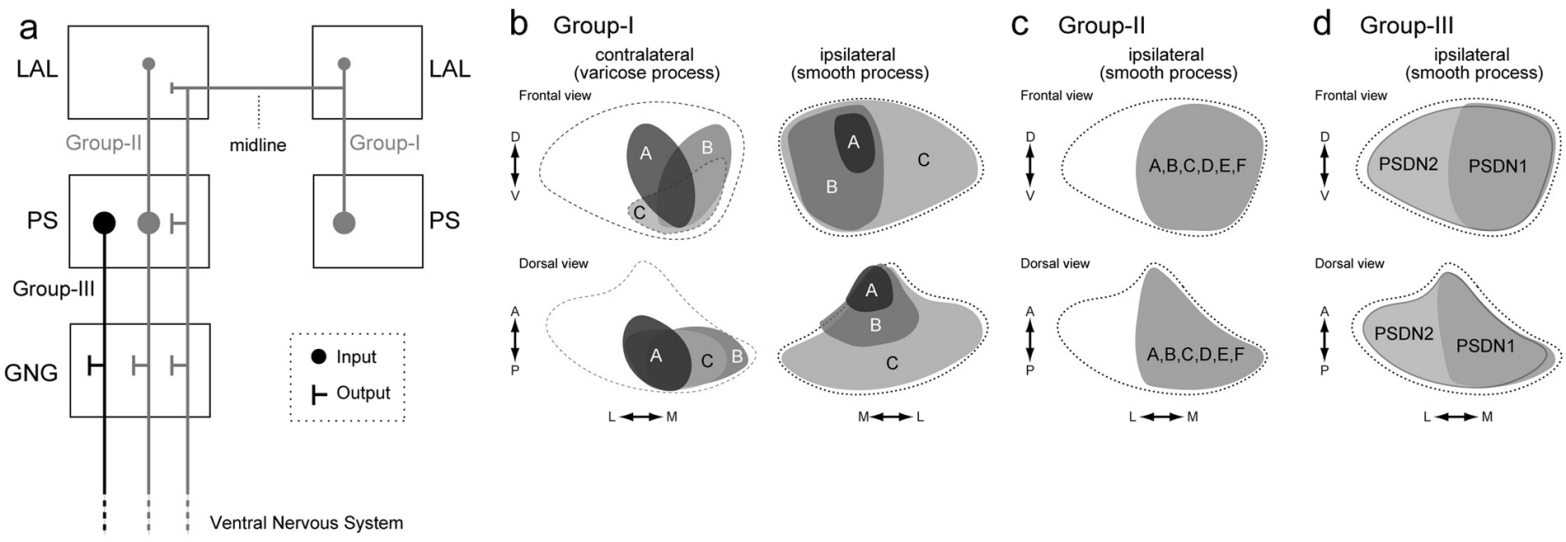
Summary of anatomical observations in descending neurons of *B. mori*. (**a**) Schematics of the descending pathway from the LAL and PS. Group-I and group-II DNs are shown in gray. Group-III DNs are shown in black. Circles and ‘T’ bars represent postsynaptic and presynaptic processes, respectively. The size of the markers reflect the relative density of innervation. All DN types have output to the GNG. **(b)** Schematics of innervation in the PS by group-I D. Ns. The PS is shown with a broken line. Frontal and dorsal views are shown. The innervation area are distinct among DN types and indicated with different color. The innervation of varicose processes in the contralateral hemisphere is biased toward the medial side of PS (*left*), which may overlap with smooth processes of group-I DNs, their contralateral counterparts. **(c)** Schematics of innervation in the PS by group-II DNs. The area of innervation for group-II DNs are similar and all DN types innervate the medial PS. Relative location of the innervation is shown in gray. This area overlaps with varicose processes of group-I DNs (panel b, *left*). (**d**) Schematics of innervation by group-III PSDNs. Both DN types innervate the medial PS.

We examined the morphology of DNs innervating the LAL in *B. mori*. We found that all DNs innervating the LAL have additional innervation in the PS (Group-I/II DNs, Figs 4, 5, 7 and 8). We also identified DNs arising from the PS that were responsive to the sex pheromone (Group-III PSDNs, Figs 9 and 10). Notably, the latter group lacks innervation to the LAL. There are at least 2 populations of DNs that are sensitive to the sex pheromone in *B. mori* (Fig. 15a).

The strategy of moth pheromone orientation is composed of two behavioral modules: (1) a surge after the stimulus onset, and (2) casting after stimulus cessation^28–32^. Flip-flop firing activity, which is toggled by pheromone input, is thought to mediate the command for zigzagging locomotion during the casting phase^14^. The LAL is thought to be crucial for generating the flip-flop neural response in the silkmoth^12,16^. A total of eight types of DNs (group-IA, IB, and IC; group-IIA, IIB, IID, and IIF; and group-III LDN2) have been identified as innervating the LAL in the present study. We revealed that these DNs have smooth processes in both LAL and PS, and there are no DNs that receive input only from the LAL. We also found group-III DNs innervating the PS, without innervation from the LAL, which exhibited an excitatory response to the sex pheromone (group-III PSDNs; Figs 9 and 10; Supplementary Fig. 12). We did not observe flip-flop activity in group-III DNs, suggesting that these DNs are involved in initial surge rather than casting. These observations provide one possibility for the organization of descending pathways for pheromone orientation: (1) group-III DNs innervating the PS mediate the initial surge, and (2) group-I/II DNs exhibiting the flip-flop response meditate casting behavior^14^ (Supplementary Fig. 15). Flip-flop DNs, however, have biphasic response properties and as the time period of the early phase is well matched to the timing of the initial surge, there is also the possibility that flip-flop DNs contribute to the signal for the initial surge.

Olfactory and visual information often interact with each other during pheromone orientation^33,34^. The silkmoth is known to use visual information for pheromone orientation^35,36^. The locomotion pattern is modified by the presence of the optic flow, suggesting the integration of pheromone and visual information. Optic flow modulates turning angular velocity during surge. DNs innervating the PS anatomically connected with lobula plate output^37,38^. The lobula complex is known to process optical flow information^39^. Such neuronal connections between the lobula plate and the PS are present in *B. mori*^16^. As group-III DNs arising from the PS demonstrate phasic responses to the sex pheromone (Figs 9 and 10; Supplementary Fig. 12), there is a possibility that these DNs mediate the visual modulation of surge response. Contrary to the surge, optic flow modulates turn duration during casting^35^, and optomotor response is absent in the casting phase, suggesting different interactions between optic flow and pheromone processing pathways for surge and casting. We found that group-I and group-II DNs innervate the PS. The dendrites of these DNs are the candidate sites for the integration of pheromone-triggered premotor information and optic flow from the PS signal during casting.

### Potential connectivity among DNs

Group-I DNs have innervation with a varicose appearance in the LAL and PS in the contralateral hemisphere to the cell body (Fig. 4). The innervation was more dense in the PS than in the LAL. The area of varicose innervation by group-I DNs is localized to the medial PS, and this area overlaps with the smooth processes of the contralateral counterparts (Fig. 15b). Furthermore, all group-II DNs have their smooth processes in the medial PS, which also overlaps with the varicose processes of group-I DNs (Fig. 15c). These anatomical observations suggest bilateral interaction of DNs via group-I DNs (Fig. 15a). Bilateral interaction between LALs in both hemispheres is thought to be important for generating the command for casting behavior. It was proposed that bilateral interneurons connecting both hemispheres mediate bilateral interaction^27,40^. There is a possibility that the pathway via group-I DNs mediate this interaction.

Group-III DNs innervating the PS responded to the sex pheromone (Figs 9 and 10; Supplementary Fig. 12), but the pathway for pheromone information to the PS is unknown. There are several possibilities. First, group-III PSDNs receive pheromone information from group-I DNs. As mentioned above, the contralateral projection of group-I DNs is localized to the medial PS, which is the group-III PSDNs major input region. Second, the PS may receive pheromone-related information from the LAL via interneurons connecting the LAL and PS^4^. Four brain regions contain neurons that selectively respond to the sex pheromone in the brain of *B. mori*: the macroglomerular complex, delta area of the inferior lateral protocerebrum, superior medial protocerebrum (SMP), and lateral accessory lobe^16^. Previous studies examines the neuroanatomy of the PS by mass staining^16^. Dye injection into the PS labels the LAL, but not the three other brain regions with pheromone responsiveness, suggesting that the PS receives pheromone information from the LAL^16^. As the mass-staining method does not label all neurons innervating the injected area, there is still a possibility that other pathway mediate the information flow. Another possibility is that the PS directly receives pheromone related information from the SMP. Although the connection between the SMP and PS has not been reported in *B. mori*, such connection is present in *Drosophila melanogaster* (e.g. fru-M-100287, TH-M-300018 & VGlut-F-200550, FlyCircuit Database^41^, http://www.flycircuit.tw/), which has a similar neuronal organization with *B. mori*^4^.

### Potential homology of neurons across species

Systematic data for the individual neuronal morphology of DNs is available in *Drosophila*^9^, enabling comparisons of neuroanatomy with *Bombyx*. Comparisons at single-cell resolution revealed similar morphological features between these genera. For example, the characteristic morphological features of group-II DNs in *Bombyx* are: (1) cell bodies belonging to the cluster are located on the anterior surface beside the anterior optic tubercle, (2) they descend the ipsilateral side of the neck connective, and (3) they innervate the PS and some of them also innervate the LAL (Fig. 7). A neuroanatomical study reports that cell groups with these morphological feature, specifically a group of ipsilaterally descending neurons with smooth processes in the PS (DNa01~DNa10)^9,42^, are present in *Drosophila*. Confocal image stacks are available on the Janelia FlyLight database (http://www.janelia.org/split-gal4). Cell groups with a similar morphological profile are present in other species, including the moth *Manduca sexta*^43^, the cricket *Gryllus bimaculatus*^44^, the locust *Schistocerca gregaria*^45^, and *Drosophila* larvae^8^.

The present study characterized the morphology of group-I DNs as follows: (1) cell bodies belonging to the cell cluster are located on the anterior surface beside the antennal lobe, (2) they descend the contralateral neck connective, and (3) they have smooth processes in the PS (Fig. 4). DNs with similar morphology have been reported in other moth species, *Manduca sexta*^43^ and *Agrotis segetum*^46^. DNs with these morphological features are present in *Drosophila* (DNb01 and DNb02) (Supplementary Fig. 16b). For example, DNb01 has morphological similarity to group-IA DN in *Bombyx*. The innervation, both in the ipsilateral and contralateral LAL, is biased toward the medial side (lower division), and the DN has varicose processes in the medial PS of the contralateral hemisphere^9^, as in group-IA DN in *Bombyx*^16^. In contrast, DNb02 has smooth innervation to both the medial and lateral sides of the PS, and the area of innervation in the LAL is small, reminiscent of group-IC in *Bombyx*. DNb02 extends smooth processes to the lateral PS, as in group-IC DN in *Bombyx* (Fig. 4). Potential homologous neurons to group-IA DNs are present in other insect orders. The VG3, a contralaterally descending neuron innervating the LAL in the locust *Schistocerca gregaria*, also has wide arborization spanning the posterior-medial protocerebrum^45^, which may correspond to the medial PS in *Bombyx*. In the ant *Camponotus obscuripes*, a descending neuron innervating the LAL also has innervation in the PS^47^. B-DC1(5), a contralaterally descending neuron innervating the LAL in the cricket *Gryllus bimaculatus* also has innervation in the protocerebrum^48^. Although the innervation of the PS has not yet been analyzed in detail, the neuron innervates an area similar to the PS^48^. These observations suggest conserved anatomical features among insects: DN innervation to the LAL always associates with the innervation to the PS, and there may be no descending signal purely originating from the LAL.

We have identified several DNs that innervate the optic lobe (Figs 11–13). DNs innervating the optic lobe are also present in flies (lobula descending neuron, DNp11)^5,9,49^. In contrast to group-III LDN1 and 2, which innervate the inner lobula, DNp11 has smooth processes in the basal layer of the outer lobula. Unlike group-III LDNs in *Bombyx*, DNp11 also innervates the antenno-mechanosensory motor center. *Drosophila* may only have a single type of DN that specifically innervates the lobula^5,9^; however, *Bombyx* has several, with at least two types of DNs innervating the lobula. Anatomical studies using backfill staining failed to find innervation to the lobula in other hemimetabolous insects such as the cockroach and cricket^6,7^. There is a possibility that group-III LDNs are a new evolutionary development in the moth and fly.

We have described the anatomical organization of DNs in the brain. The neuroanatomy discussed here contributes to the investigation of neural mechanisms underlying insect behavior. The functional difference between these two pathways characterized in the present study, i.e., LAL + PS output vs. pure PS output is of interest (Fig. 15a). Future studies should examine differences in the function and physiology for these pathways.

## Materials and Methods

### Experimental animals

*B. mori* (Lepidoptera: Bombycidae) were reared on an artificial diet (Silk Mate 2S and PS; Nosan Bio Department, Yokohama, Japan) at 26 °C and 60% relative humidity, under a long-day photoperiod regime (16/8-h light/dark). Animals were used within 2–7 days of eclosion.

### Olfactory stimulation

Synthetic (E,Z)−10,12-hexadecadien-1-ol (bombykol), the principal pheromone component of *B. mori*, with a purity of >99%, as confirmed by gas chromatography, was dissolved in high-performance liquid chromatography-grade n-hexane. The odorant (5 μl of solution) was applied to a piece of filter paper (1 × 2 cm) and inserted into a glass stimulant cartridge with a 5.5-mm-tip diameter. The distance between the filter paper and the cartridge exit was approximately 7 cm. We applied 10 ng of bombykol to the filter paper, which corresponds to the amount that induces transient bursting activity in projection neurons in the AL and reliably triggers behavioral responses. Air or the odor stimulus was applied to either side of the antenna, and the exit of the cartridge was positioned 1.5 cm from the antennae. Compressed pure air was passed through a charcoal filter into the stimulant cartridge, and each stimulus was applied at a velocity of 500 mL min^−1^ (approximately 35 cm s^−1^), nearly the same as the flow speed produced when moths flap their wings. The moths were exposed to the odor for 200 or 500 ms, after which an exhaust tube was placed on the opposite side of the stimulant cartridge and the odor was removed (inner diameter, 4.5 and 15 cm from the antennae; ~55 cm s^−1^). All stimulant cartridges were sealed with a Teflon sheet, stored at −20 °C, and brought to room temperature prior to the recording session.

### Intracellular recording and staining

The staining procedure was conducted as previously described^50^. After cooling (4 °C, ∼30 min) to induce anesthesia, the abdomen, legs, wings, and dorsal side of the thorax were removed. The moth was fixed in a plastic chamber, and its head was immobilized using a notched plastic yoke slipped between the head and thorax. The brain was exposed by opening the head capsule and removing the large tracheae, and the intracranial muscles were removed to eliminate brain movement. The AL was surgically desheathed to inserte the microelectrode.

Filamented glass capillaries (TW100F-3; World Precision Instruments, Sarasota, FL, USA) were pulled on a micropipette puller (P-97 or P-2000; Sutter Instruments, Novato, CA, USA) and filled with 5% Lucifer yellow CH (LY) solution (Sigma, St. Louis, MO, USA) in distilled water or 1 M lithium chloride for staining neurons. The resistance of the electrodes was ∼60–300 MΩ. The electrodes were inserted using a micromanipulator (Leica Microsystems, Wetzlar, Germany), and a silver ground electrode was placed on the head cuticle. The brain was superfused with saline solution containing 140 mM NaCl, 5 mM KCl, 7 mM CaCl_2_, 1 mM MgCl_2_, 4 mM NaHCO_3_, 5 mM trehalose, 5 mM N-tris(hydroxymethyl)methyl-2-aminoethanesulfonic acid (TES), and 100 mM sucrose (pH 7.3). The incoming signals were amplified (MEZ-8300; Nihon Kohden, Tokyo, Japan), monitored with an oscilloscope (VC-10; Nihon Kohden), and recorded on a DAT recorder (RD-125T; TEAC, Tokyo, Japan) at 24 kHz. The acquired signals were stored in a computer using an A/D converter (PCI-6025E; National Instruments, Austin, TX, USA).

We stained each neuron using an iontophoretic injection of LY with a constant hyperpolarizing current (approximately −1 to −5 nA) for 1–3 min. After staining, the brain was superfused with saline solution containing 200 mM sucrose. Brains were fixed in 4% paraformaldehyde for 1–24 hours at 4 °C. Brains were then dehydrated with 70%, 80%, 90%, 95%, and 100% ethanol (10 min in each), and cleared in methylsalicylate for at least 30 min.

### Backfill labelling

The ventral part of the neck was dissected to expose the neck connective. The nerve was stained by filling it with saturated LY dissolved in distilled water from the cut end of the neck connective overnight at 4 °C. The neck connective was placed in a pool made with Vaseline on a cover glass. To only stain one side, one of the neck connectives was damaged using forceps. After backfilling, the head was immediately removed. The brain was dissected from the head, and then dissected, fixed, dehydrated, and cleared, as described above.

### Immunolabeling

After dissection of the brains from the head capsules, they were fixed in 4% paraformaldehyde in 0.1 M sodium phosphate-buffered saline (PBS, pH 7.4) overnight at 4 °C. The brains were washed several times in 0.1 M PBS containing 0.2% Triton X-100 (PBST, pH 7.4) and incubated with 5% normal donkey serum (NDS) (D9663; Sigma, St. Louis, MO) or normal goat serum (NGS) (D9023; Sigma) in PBST (PBST-NDS or -NGS) for 3 hours at room temperature. Then, they were incubated with the anti-synaptotagmin antibody (1:30) for 2 days at 4 °C. The brains were washed in PBST and incubated with the secondary antibody diluted 1:200 in PBST-NDS or -NGS overnight at 4 °C. After washing, the brains were dehydrated through an ascending ethanol series and cleared in methyl salicylate.

### Imaging

Each stained neuron was visualized using a confocal imaging system (LSM510; Carl Zeiss, Jena, Germany) with ×40 (numerical aperture = 1.0) objective. LY-stained neurons were examined at a 458-nm excitation wavelength with a long-pass emission filter (>475 nm) in whole mounts. In some cases, we detected an autofluorescence signal using a HeNe laser at 543 nm and measured instead with a 560-nm long-pass filter. Serial optical sections were acquired at 0.7- or 1.4-μm intervals throughout the depth of the neuron, and three-dimensional reconstructions of the labeled neurons were created from these sections.

### Anatomical nomenclature

Regarding the neuroanatomical terminology, we followed the brain nomenclature proposed by Ito *et al*.^24^. We updated the use of terminology in *B. mori*: we used the term “esophagus” for “oesophagus,” “gnathal ganglion” for “suboesophageal ganglion”, and “antennal-lobe tract” for “antenno-protocerebral tract.”

We previously identified three types of DNs in the silkmoth brain^12^ (Fig. 1). There are two groups of cell bodies on the anterior brain surface (Fig. 1a). Dorsal and ventral groups are termed “group-I” and “group-II”, respectively. Other types whose cell bodies were located on the posterior surface were classified as “group-III” (Fig. 1b). Group-I and group-II DNs descend via the contralateral and ipsilateral neck connective, respectively (Fig. 1d). Group-III contains both ipsilateral and contralateral DNs.

The anatomical border on the anterior side of the LAL is relatively well-defined and the posterior side was defined by the depth of the saddle point of the lateral antennal-lobe tract in most cases^27^. Adjacent to the LAL is a small region called the VPC (Supplementary Fig. 1). We have not yet identified any local interneurons confined within the VPC and some LAL interneurons have additional branches within the VPC^27^. As no clear anatomical border is present between the LAL and VPC. The saddle point of the lateral antennal-lobe tract was used to discriminate the LAL and VPC. The PS is an unstructured neuropil, which is located posterior to the VPC. No clear anatomical boundary is available except for the posterior border, which is defined by the brain surface. These three neuropils, LAL, VPC, and PS, are arranged serially and do not have clear anatomical boundaries (Fig. 1e).

Abbreviations used is summarized in Supplementary Table 2.

### Data analysis

The segmentation and volume rendering of neurons and neuropils were performed with AMIRA 6.2 (FEI, Hillsboro, OL, USA). For images of single neuron morphology, masked images were used for visualization. We performed segmentation of individual neurons in confocal stacks. We first detected the signal with the Amira “Interactive Thresholding” function. We subsequently corrected any false detection by manual tracing. Using this image as a mask, we obtained the final masked images shown in the figures using a custom-made program written in MATLAB and the image processing toolbox (MathWorks, Natick, MA, USA). Maximum intensity projection images were prepared with ImageJ (National Institutes of Health, Bethesda, MD, USA)^51^. The contrast and brightness of images were modified in Image J. Figures were prepared in Adobe Illustrator CS (Adobe Systems, San Jose, CA, USA).

## Electronic supplementary material

Z stack images of confocal microscopy data of backfill labeling

Supporting Online Material
