# Supplementary material for: Descending neurons from the lateral accessory lobe and posterior slope in the brain of the silkmoth Bombyx mori
Source: Sci Rep. 2018 Jun 25;8:9663. doi: 10.1038/s41598-018-27954-5 (PMC6018430; doi:10.1038/s41598-018-27954-5)
Supplement: Supplementary file 2 — Supporting Online Material [file 41598_2018_27954_MOESM2_ESM.docx]

**Scientific Reports**

**Supplementary Information**

**Descending neurons from the lateral accessory lobe and posterior slope in the brain of the silkmoth *Bombyx mori***

Shigehiro Namiki, Satoshi Wada, Ryohei Kanzaki

Research Center for Advanced Science and Technology, The University of Tokyo, 4-6-1 Komaba, Meguro, Tokyo 153-8904, Japan. Correspondence should be addressed to S.N. (email: [namiki@rcast.u-tokyo.ac.jp](mailto:namiki@rcast.u-tokyo.ac.jpr))

16 Supplementary Figures, 2 Supplementary Tables, 1 Supplementary Video


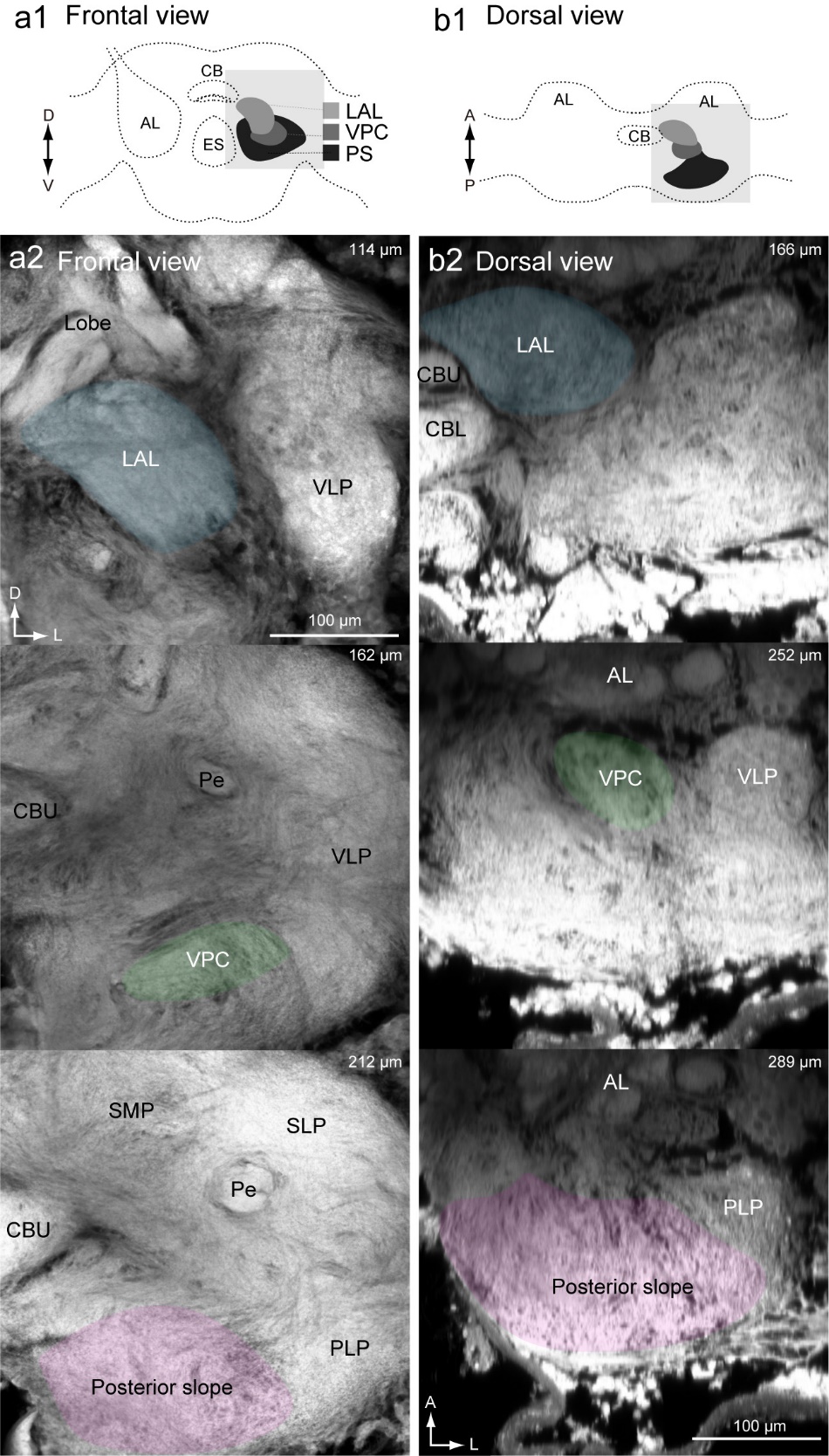


**Supplementary Figure 1. The schematics of the location of brain neuropils, lateral accessory lobe (LAL), ventral protocerebrum (VPC) and posterior slope (PS).** Schematics in frontal (**a1**) and dorsal views are shown (**b1**). Confocal stacks for brain tissue labeled with anti-synaptotagmin antibody are shown (**a2** for frontal view, **b2** for dorsal view). Brain regions, LAL, VPC and PS are labeled with blue, green and magenta, respectively. The depth from the anterior surface are shown in the *top-right*. AL, antennal lobe; CBL, central body lower division; CBU, central body upper division; Pe, pedunculus of the mushroom body; PLP, posterior lateral protocerebrum; SLP, superior lateral protocerebrum; SMP, superior medial ptorocerebrum.


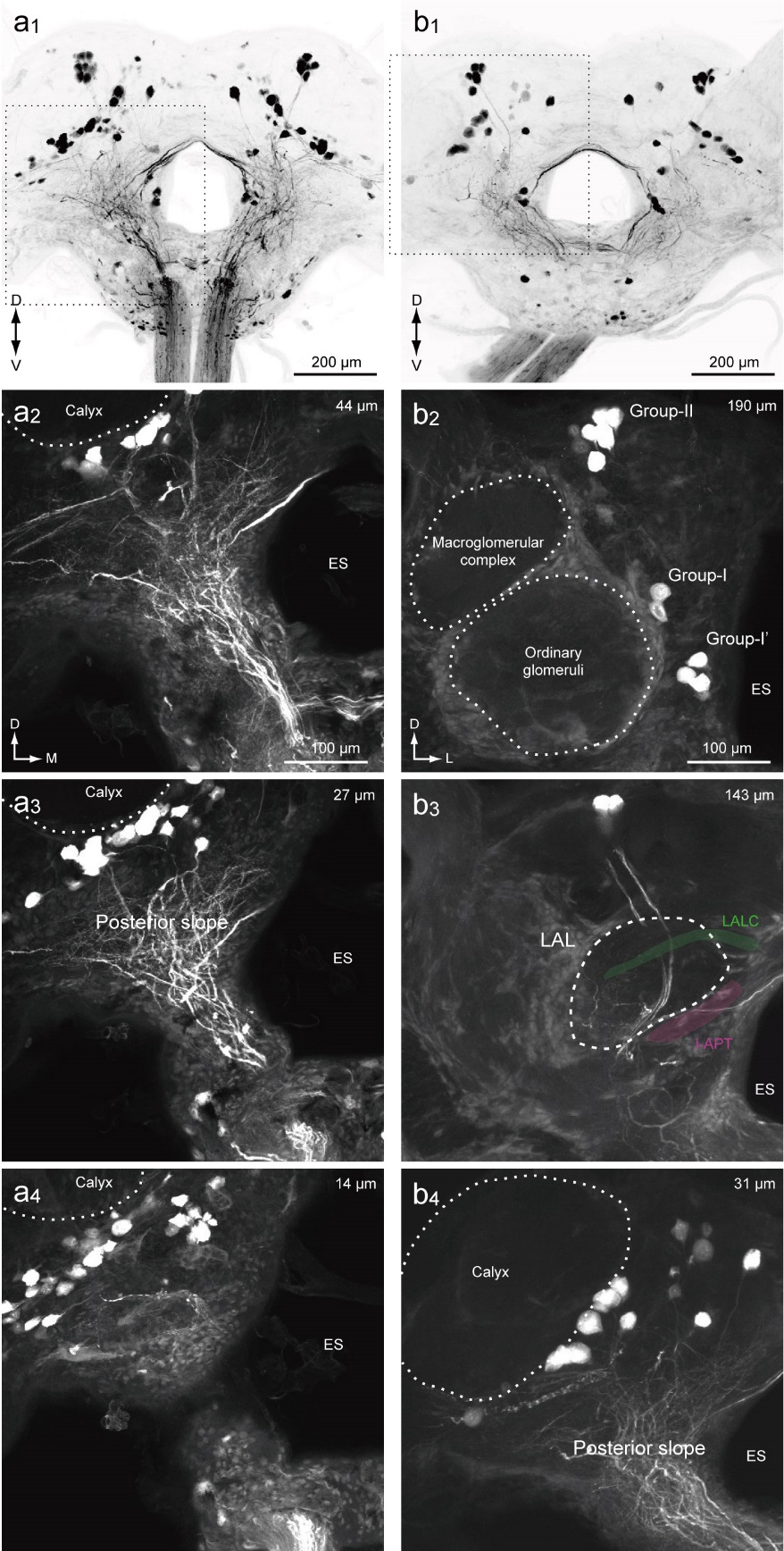


**Supplementary Figure 2. Brain samples with backfill staining.** Maximum intensity projection for confocal stacks of staining results (a1, b1) and confocal section at different depth are shown (a2-4, b2-4). The depth from the posterior brain surface are shown in the *top-right*. The neuropil regions are shown with broken lines. ES, esophagus; LALC, lateral accessory lobe commissure; l-ALT, lateral antennal-lobe tract.


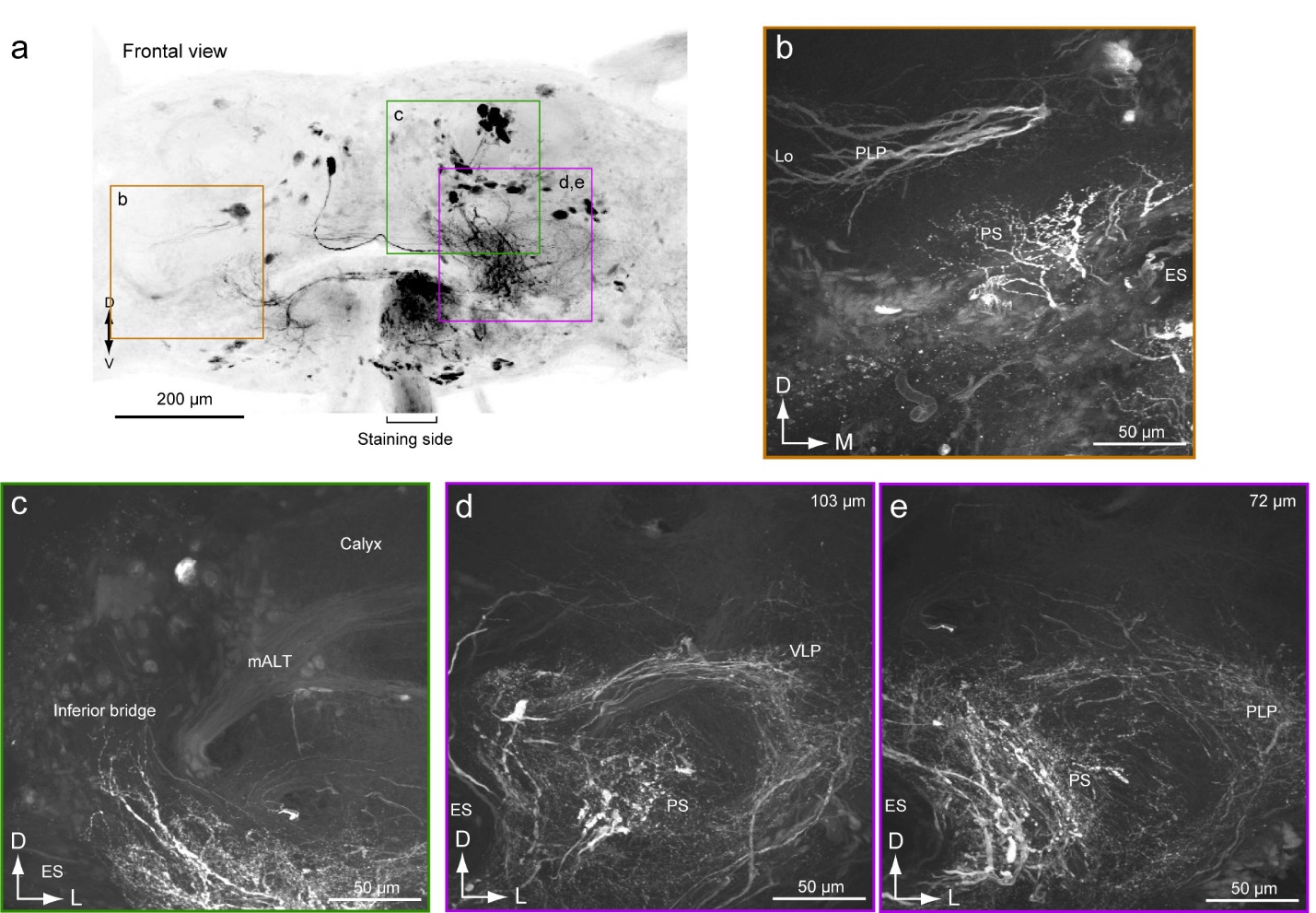


**Supplementary Figure 3. Backfill staining from one side of the neck connective.** (**a**) Whole brain image of the sample. (**b-e**) High resolution images for the area shown in (a). Posterior lateral protocerebrum (PLP) and posterior slope (PS) of the contralateral hemisphere (b), inferior bridge (c), and PS, ventral lateralprotocerebrum (VLP) and PLP of the ipsilateral hemisphere are shown (d,e). The specimen is the same with the one shown in Figure 3. ES, esophagus; m-ALT, medial antennal-lobe tract; Lo, lobula.


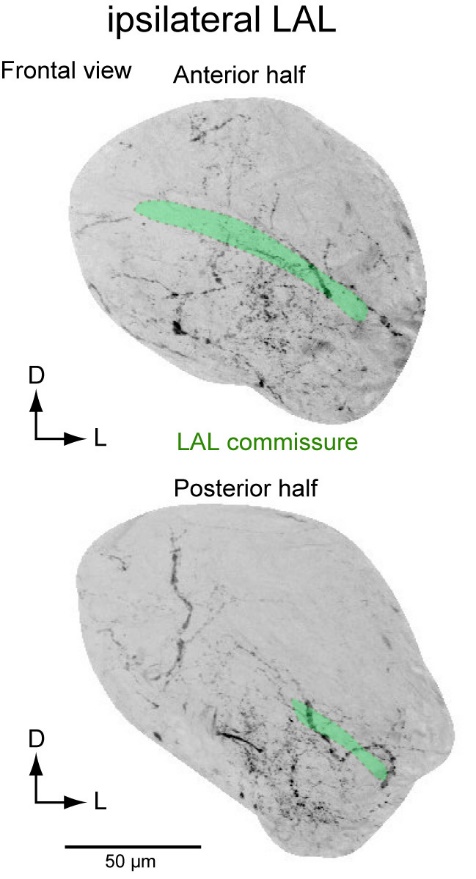


**Supplementary Figure 4. Innervation in the LAL in backfill sample stained by one side of the neck connective.** Innervation in the anterior (*top*) and posterior half of the LAL are shown (*bottom*). The position of the LAL commissure is shown in green. The volume of the LAL was used as a mask to show the innervation within the LAL. As in the sample of backfilling from both sides of the connective (Fig. 2d), lower division of the LAL contained more innervation than the upper division. The specimen is the same with the one shown in Figure 3.


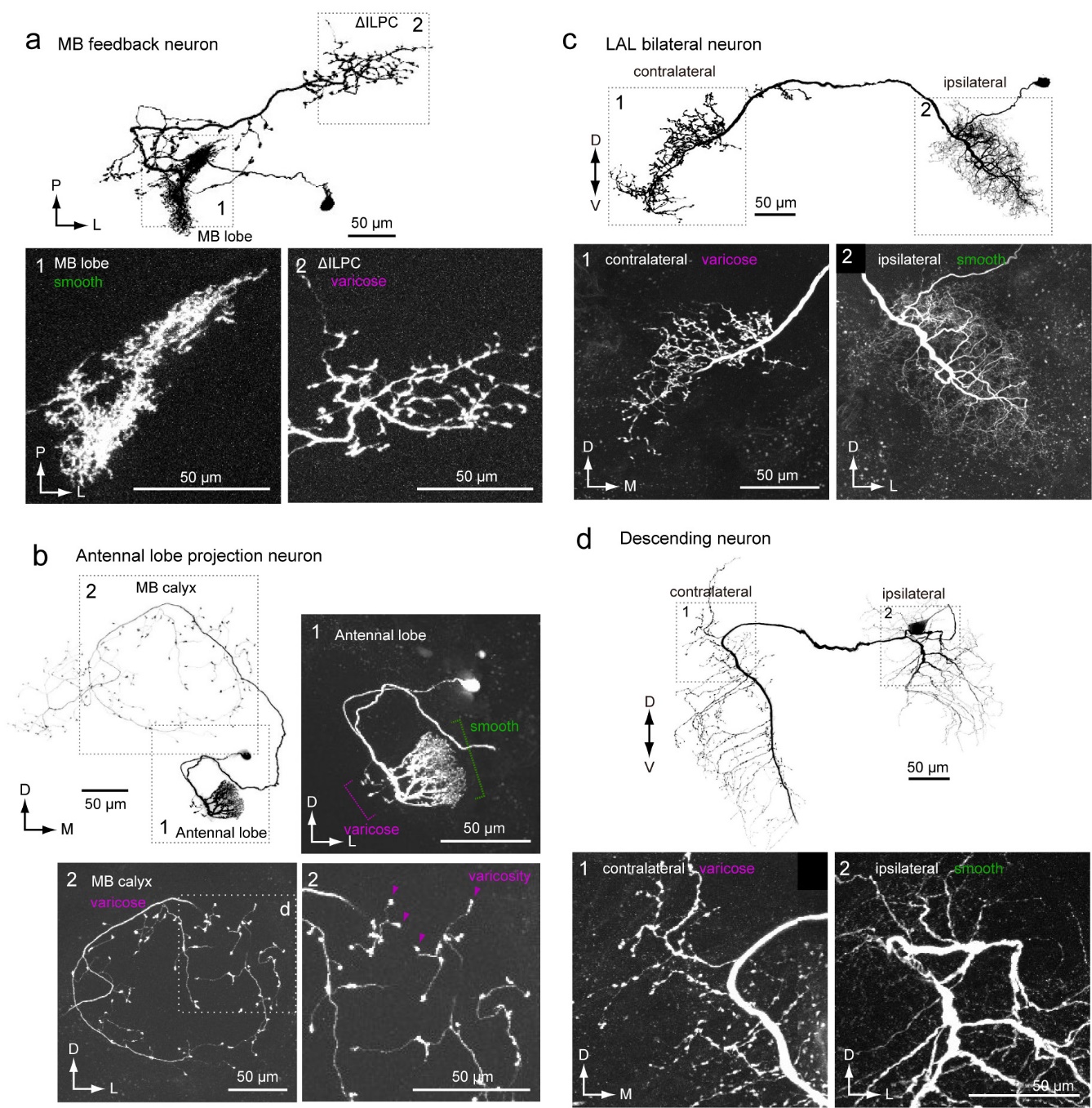


**Supplementary Figure 5. Terminal morphology of neuronal innervation. (a)** Morphology of a mushroom body (MB) feedback neuron. The innervation in the medial lobe of the mushroom body shows smooth appearance (panel 1). Innervation in the delta area of the inferior lateral protocerebrum (ΔILPC) shows varicose appearance (panel 2). **(b)** Morphology of an antennal lobe projection neuron. Most of the innervation in the antennal lobe show smooth appearance (green in top-right panel). The innervation in the mushroom body calyx show varicose appearance (bottom panels). Individual varicosities are indicated by arrowheads (magenta). **(c)** Morphology of a lateral accessory lobe (LAL) bilateral neuron. Innervation in the hemisphere ipsilateral to the cell body position show smooth appearance (panel 2), whereas the innervation in the hemisphere contralateral to the cell body show varicose appearance (panel 1). **(d)** Morphology of a descending neuron. Innervation in the hemisphere ipsilateral to the cell body show smooth appearance (panel 2), whereas the innervation in the hemisphere contralateral to the cell body show varicose appearance (panel 1). In all cases, neurite with smooth appearance was closer to the cell body, than those with varicose appearance (a-d). In case of neurons with bilateral innervation, innervation in the ipsilateral side to the cell body show smooth appearance whereas those in the contralateral side show varicose appearance (c, d). Original data are taken from Namiki et al. 2014 Nat Commun 5:5919 for a & c, Namiki & Kanzaki 2011 J Comp Neurol 519:3367 for b.


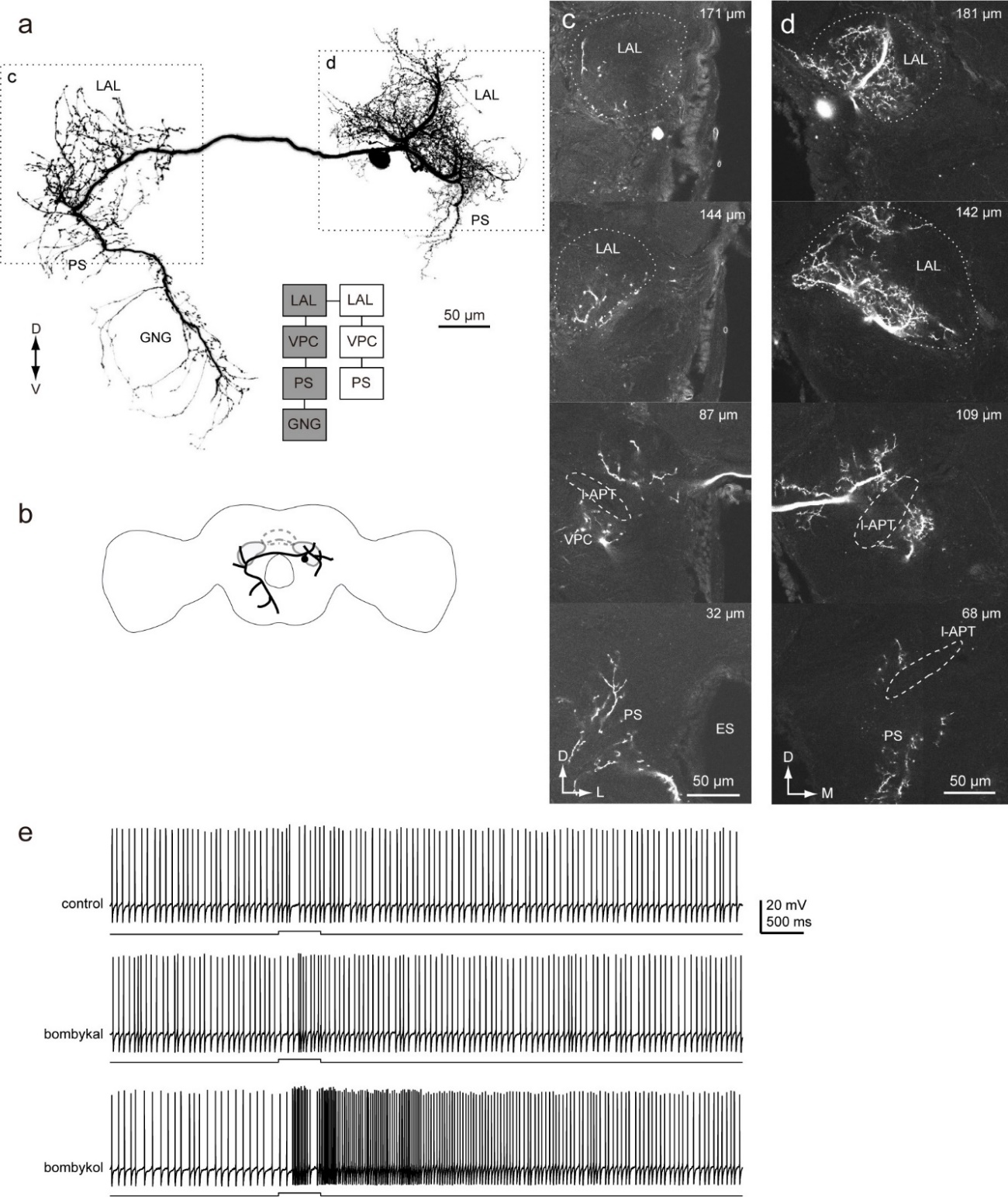


**Supplementary Figure 6. Morphology of group-IA descending neuron.** (**a**) Frontal view of group-IA DN. Inset shows a schematics of neurite morphology. White and gray boxes represent smooth and varicose appearance are dominant in the brain region. (**b**) Schematic of neuronal innervation shown in (a). (**c,d**) Confocal stacks in left and right hemispheres. Innervation to the posterior slope (PS) is observed in both sides. The lateral accessory lobe (LAL) and lateral antennal lobe tract (l-ALT) are shown in broken lines. The depth from the posterior brain surface are shown in the *top-right*. (**e**) Odor response of the neuron. The neuron exhibited weak response to bombykal and bi-phasic excitatory response to bombykol. ES, esophagus; GNG, gnathal ganglion; l-ALT, lateral antennal-lobe tract.


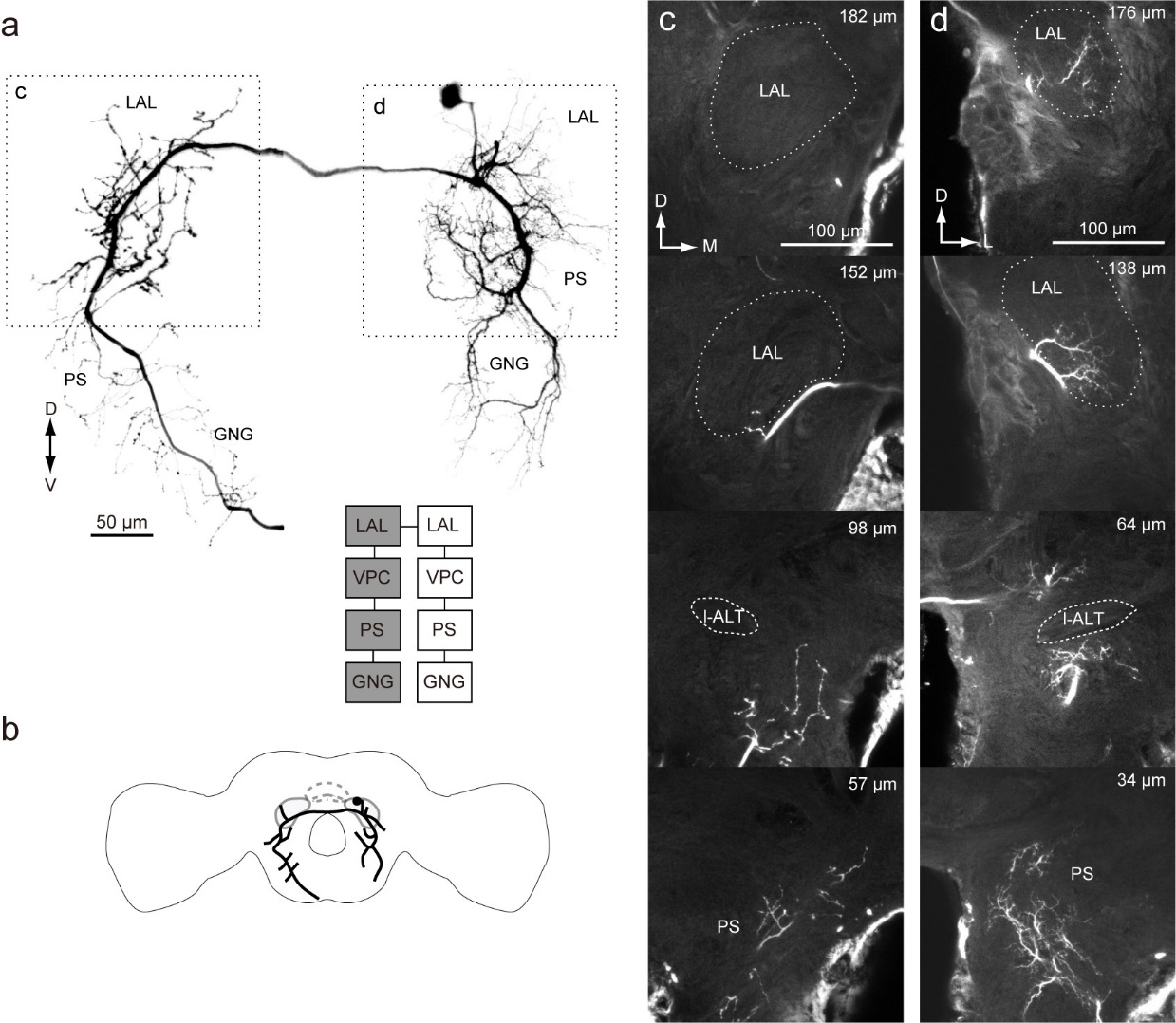


**Supplementary Figure 7. Morphology of group-IB descending neuron.** (**a**) Frontal view of group-IB DN. Inset shows areal connectivity of the neuron. White and gray boxes represent the presence of smooth and varicose processes. (**b**) Schematic of neuronal innervation shown in (a). (**c,d**) Confocal stacks in left and right hemispheres. Innervation to the posterior slope (PS) is observed in both sides. Bifurcated branches enter to the GNG in the ipsilateral hemisphere. The depth from the posterior brain surface are shown in the *top-right.* GNG, gnathal ganglion; LAL, lateral accessory lobe; l-ALT, lateral antennal-lobe tract.


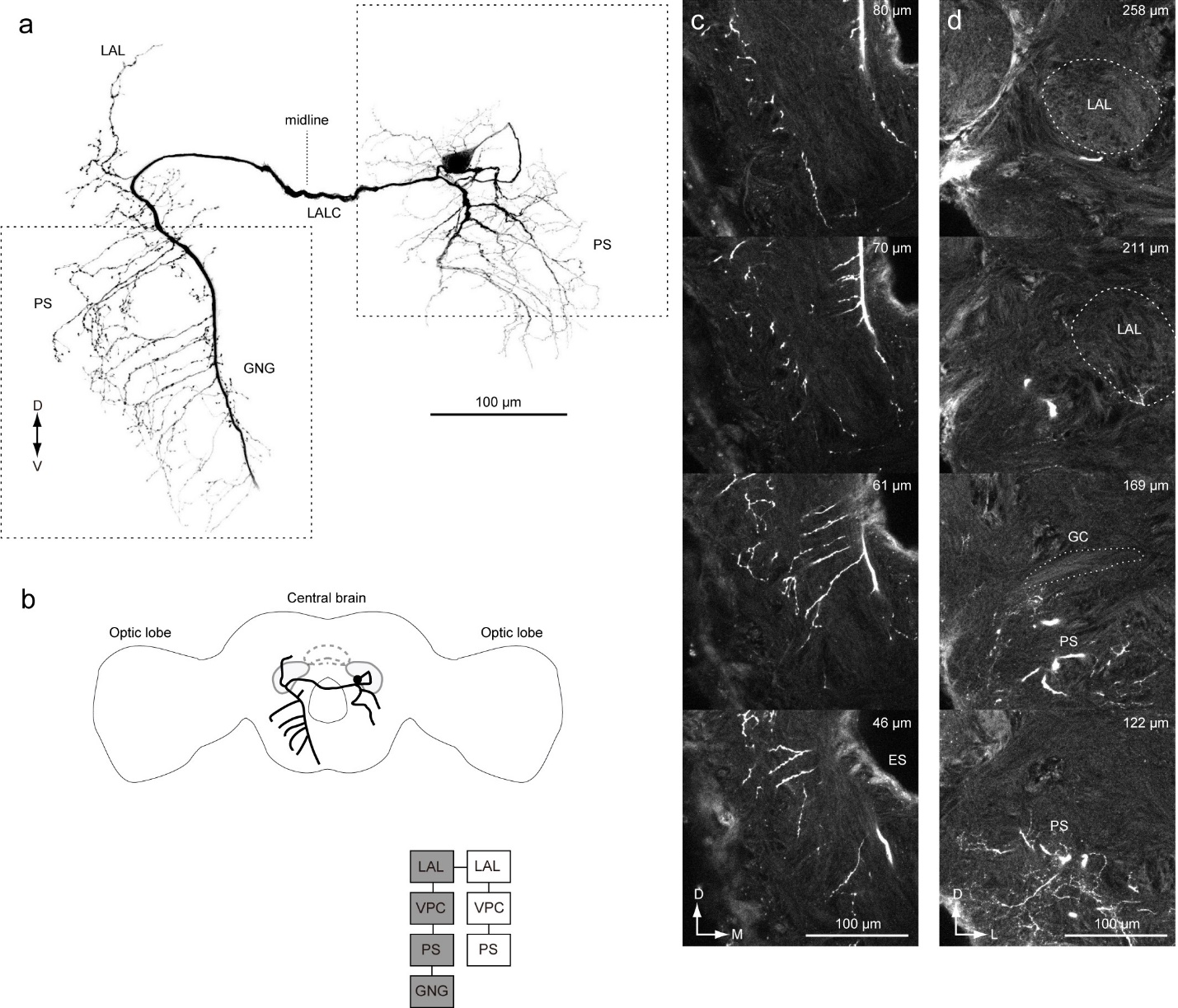


**Supplementary Figure 8. Morphology of group-IC descending neuron.** (**a**) Frontal view of group-IC DN. Inset shows areal connectivity of the neuron. White and gray boxes represent the presence of smooth and varicose processes. (**b**) Schematic of neuronal innervation shown in (a). (**c,d**) Confocal stacks in left and right hemispheres. A few branches enters in the lateral accessory lobe (LAL) on both sides. Innervation to the posterior slope (PS) was observed in both sides. The neuron innervates entire field in the PS of the ipsilateral hemisphere. The depth from the posterior brain surface are shown in the *top-right*. ES, esophagus; GC, great commissure; ES, esophagus; GC, great commissure; GNG, gnathal ganglion; LALC, lateral accessory lobe commissure.


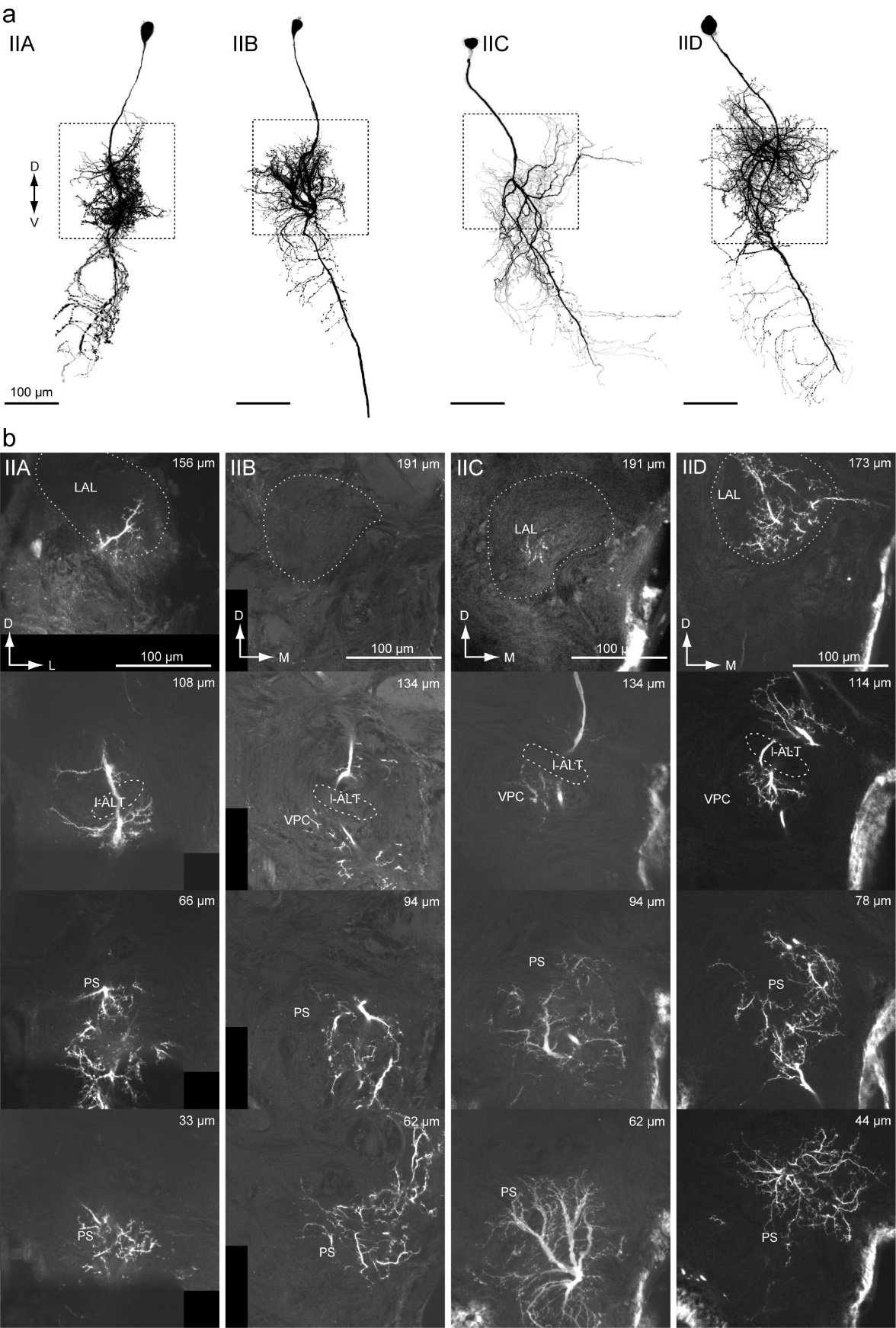


**Supplementary Figure 9. Morphology of group-II descending neurons.** (**a**) Frontal view of the maximum intensity projection image of group-II DNs. (**b**) Confocal stacks of the neuronal innervation. The imaging area is shown by a broken line shown in (a). Each DN type has innervation in the medial side of the posterior slope (PS). The depth from the posterior brain surface are shown in the *top-right*. LAL, lateral accessory lobe; l-ALT, lateral antennal lobe tract; VPC, ventral protocerebrum.


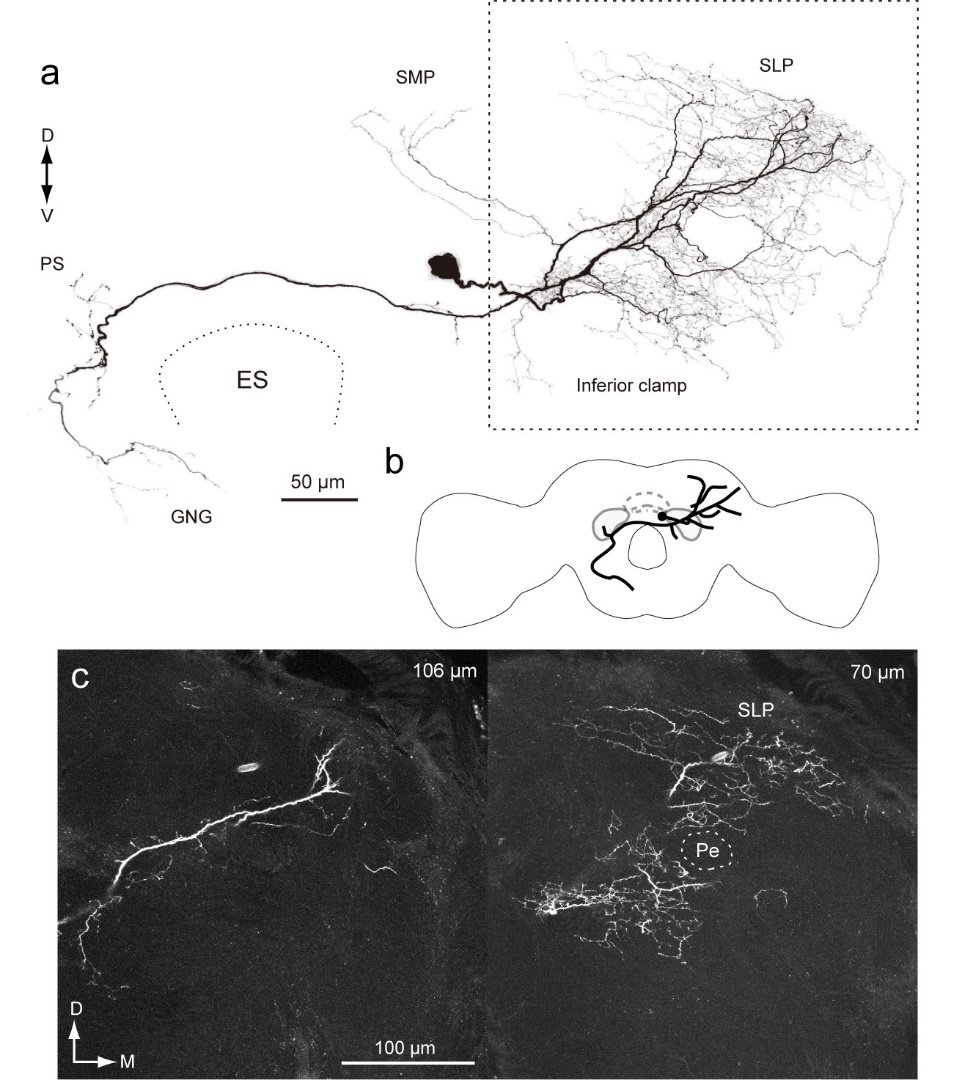


**Supplementary Figure 10. A descending neuron (DN) innervating dorsal protocerebrum**. (**a**) Frontal view of maximum intensity projection of the DN. The DN has smooth process in the superior lateral protocerebrum (SLP), superior medial protocerebrum (SMP) and inferior clamp of the ipsilateral hemisphere and varicose process in the posterior slope (PS) and gnathal ganglion of the contralateral hemisphere. (**b**) Schematics of the neuronal innervation. (**c,d**) Confocal stacks of the neuronal innervation in the protocerebrum. The depth from the posterior brain surface is shown in the *top-right*. Pe, pedunculus of the mushroom body.


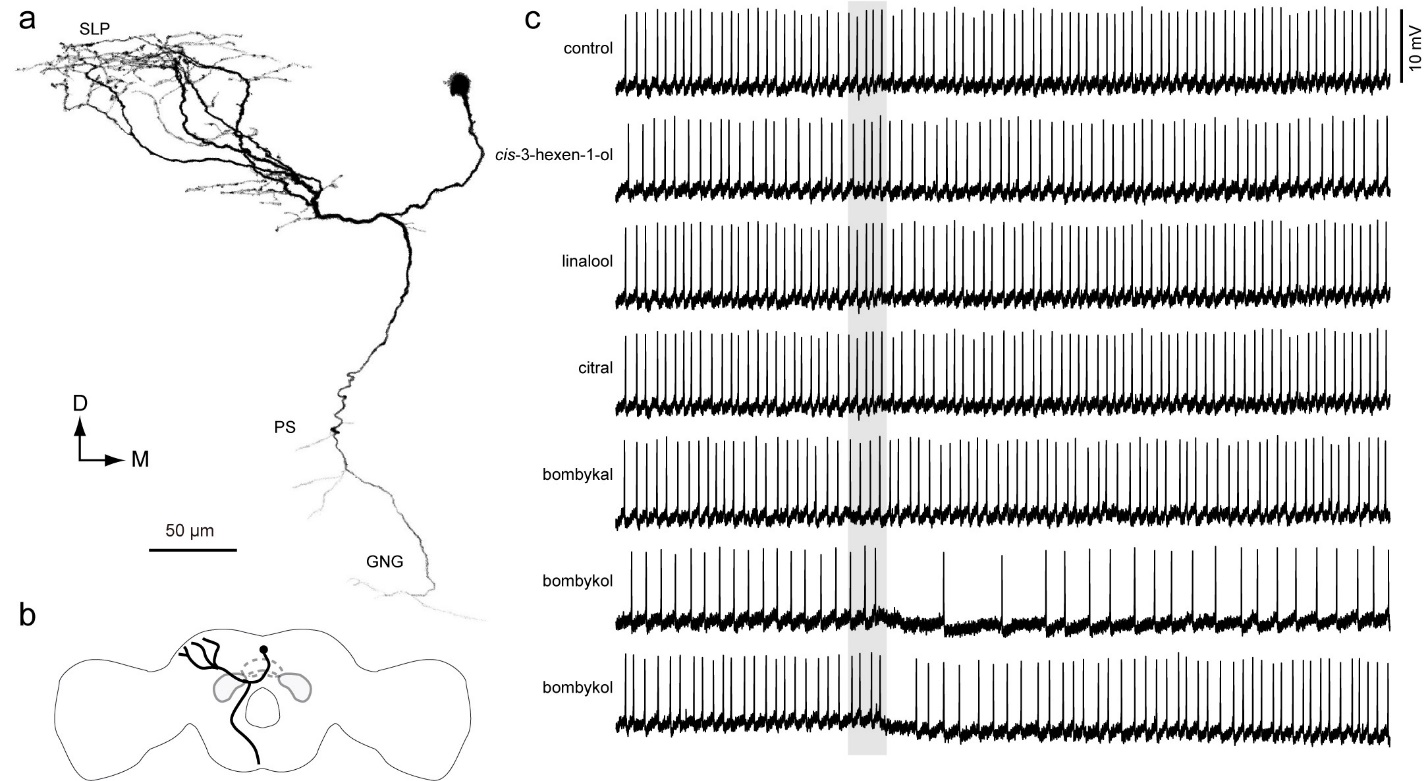


**Supplementary Figure 11. Morphology of a ipsilateral descending neuron innervating the superior lateral protocerebrum (SLP).** (**a**) Frontal view of the maximum intensity projection of neuronal innervation in the brain. The neuron has smooth process in the SLP and varicose process in the posterior slope (PS) and gnathal ganglion (GNG) of the ipsilateral hemisphere. (**b**) Schematic of the neuronal innervation. (**c**) Response property of the neuron. The neurons exhibited inhibitory response to the sex pheromone.


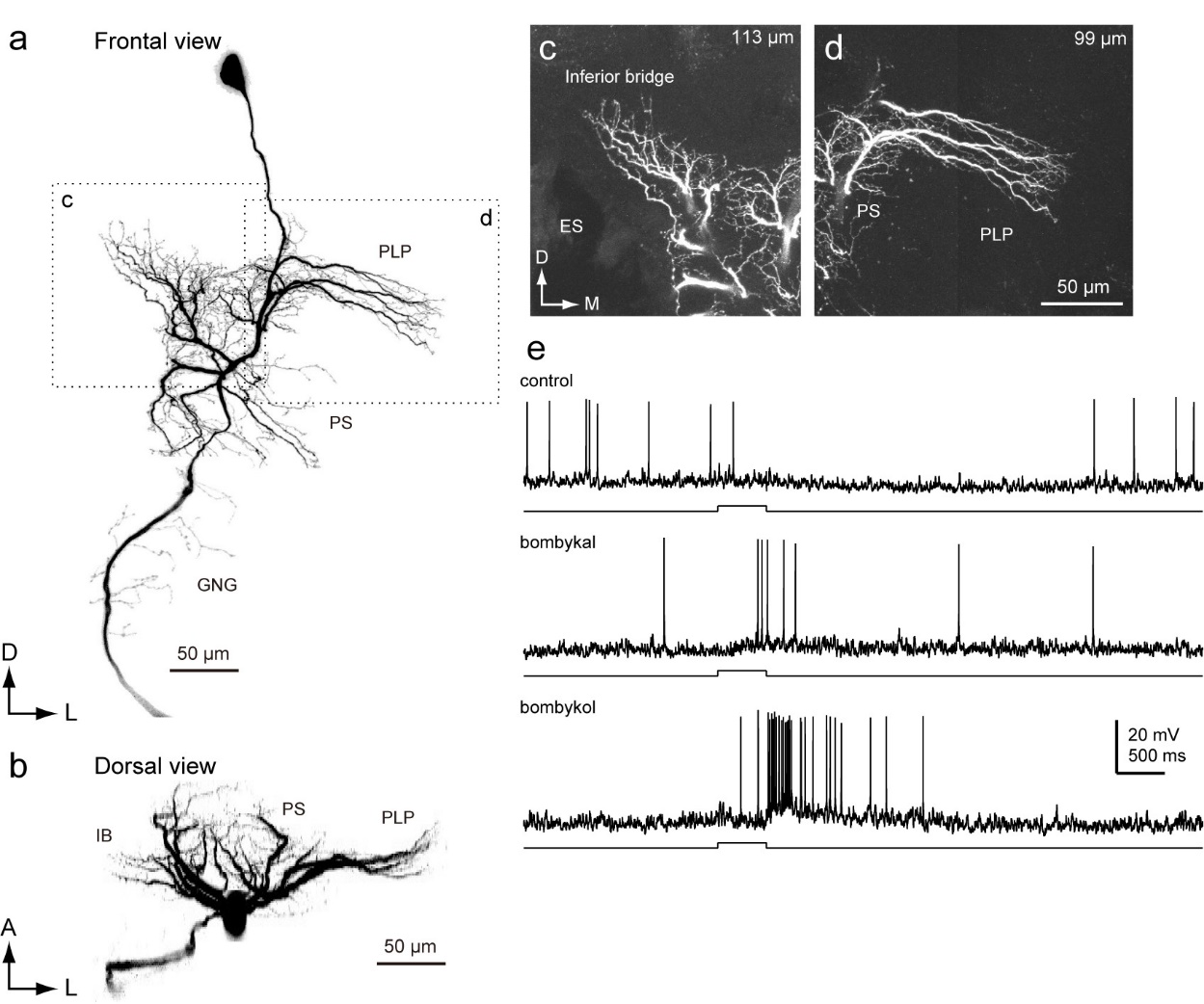


**Supplementary Figure 12. Morphology of a descending neuron innervating the posterior slope (PS).** (**a,b**) Frontal and dorsal views of maximum intensity projection of the DN innervation in the brain. The neuron has smooth process in inferior bridge (IB), posterior lateral protocerebrum (PLP) and PS, and varicose process in the gnathal ganglion (GNG). (**c,d**) Confocal stacks for the DNs shown in (a). The depth from the posterior brain surface is shown in the *top-right*. (**e**) Response property of the neuron. The neuron exhibited excitatory response to the exposure of bombykol. The morphology and physiology is similar with the neuron shown in Figure 9. ES, esophagus.


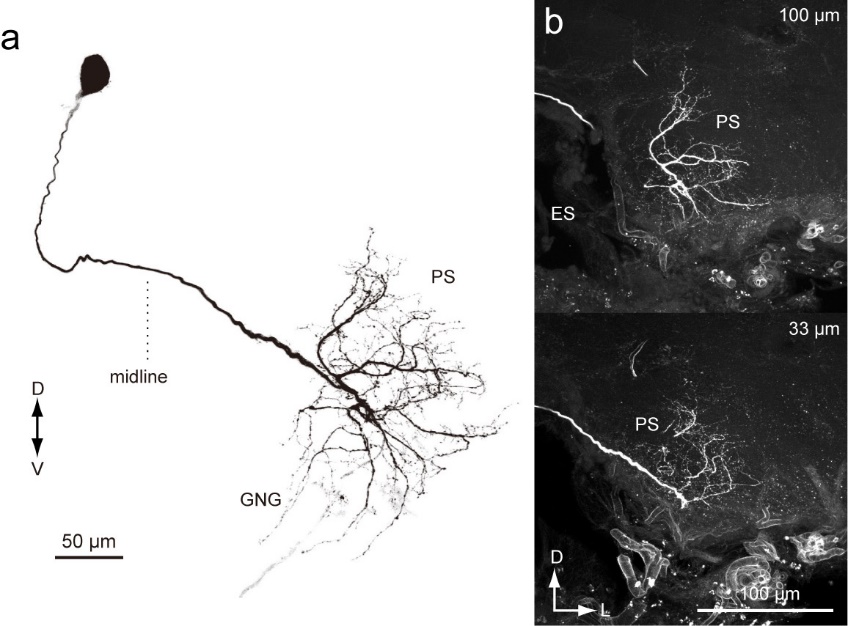


**Supplementary Figure 13. Morphology of a descending neuron innervating the posterior slope (PS).** (**a**) Maximum intensity projection of the DN innervation in the brain. The neuron has smooth process in the PS and gnathal ganglion (GNG), and varicose process in the GNG. (**c**) Confocal stacks for the DN shown in (a). The depth from the posterior brain surface is shown in the *top-right*. The neuron lacks innervation in the LAL. The morphology and physiology is similar with the neuron shown in Figure 10. ES, esophagus.


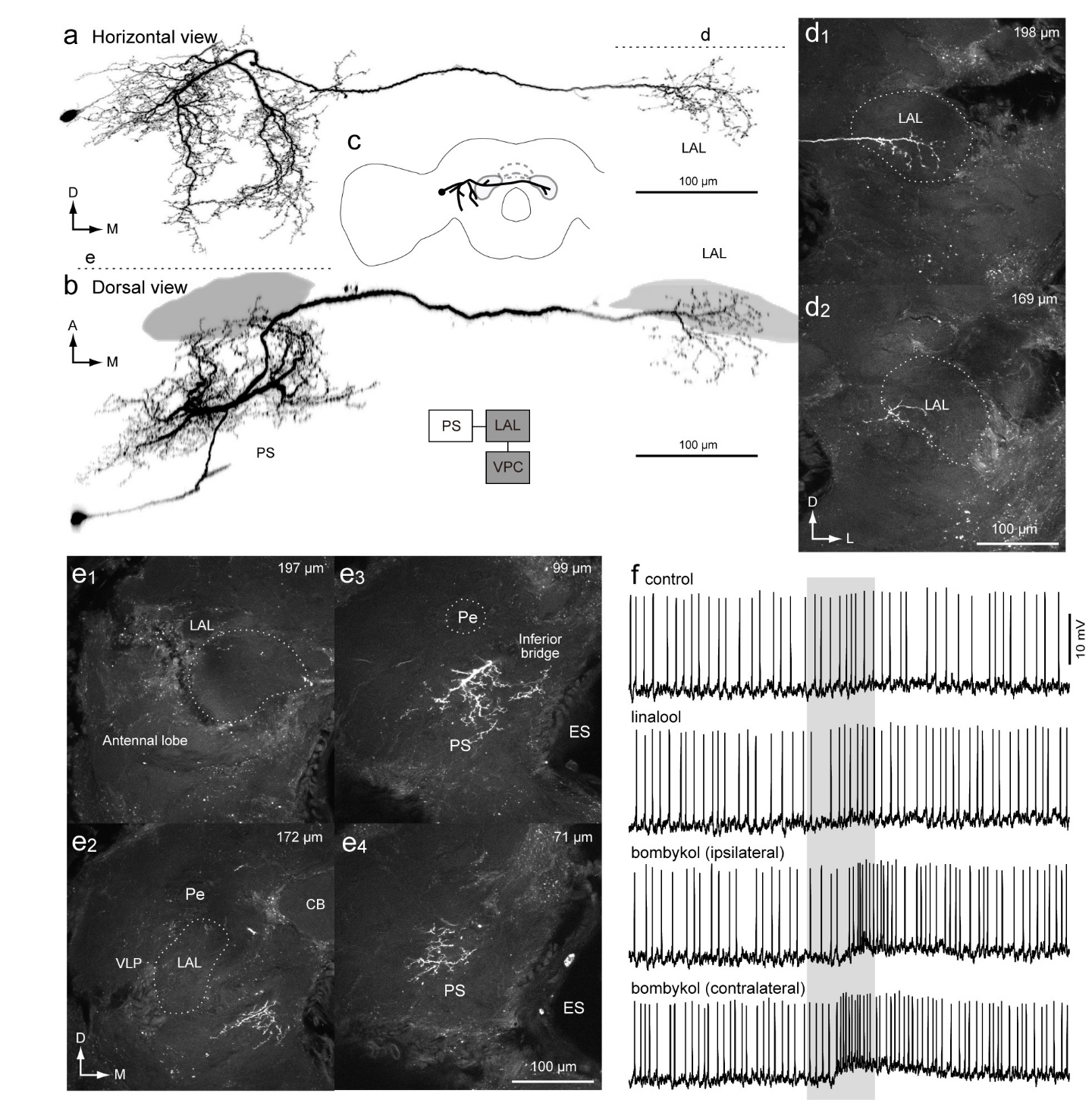


**Supplementary Figure 14. Morphology of a bilateral neuron connecting the PS and LAL.** (**a,b**) Frontal and dorsal views of maximum intensity projection of the neuronal innervation in the brain. The neuron has wide field innervation with smooth appearance in the PS of the ipsilateral hemisphere and varicose process in the LAL of the contralateral hemisphere. The volume of the LAL is shown with gray in panel b. Inset shows schematics of the innervation area of the neuron. Outlined and gray boxes represent the presence of smooth and varicose process. (**c**) Schematic of the neuronal innervation. (**d**) Confocal stacks for innervation in the contralateral hemisphere of the neuron shown in (a). The depth from the posterior brain surface is shown in the *top-right*. (**e**) Confocal stacks for innervation in the ipsilateral hemisphere of the neuron shown in (a). The neuron does not innervate the LAL of the ipsilateral hemisphere. (**f**) Response property of the neuron. The neuron exhibited excitatory response to exposure to bombykol. Gray box represents the time period of stimulation.


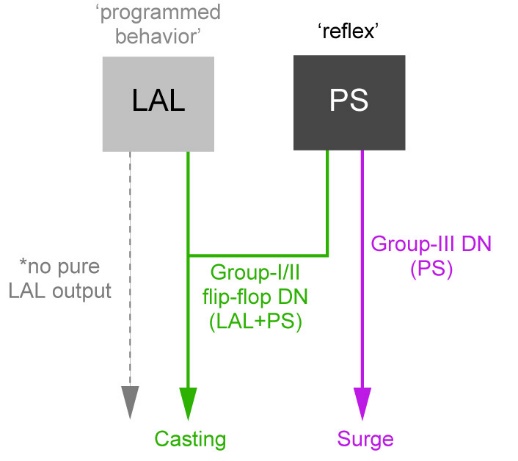


**Supplementary Figure 15. Schematics of hypothetical view of descending pathways for pheromone orientation.** Group-I and II DNs signal the command for zigzagging program during casting (green) and group-III DNs signal the command for initial surge response (magenta). The DNs which receive input only from the LAL have not been identified thus far (broken line).


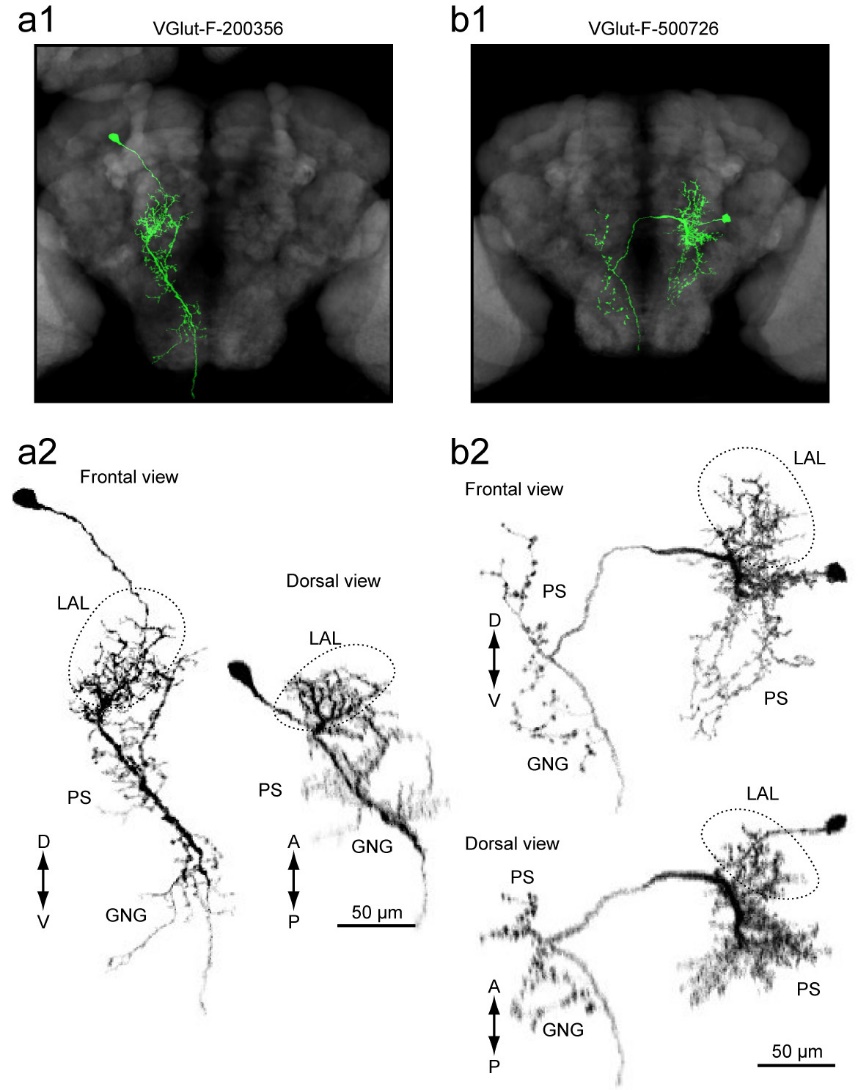


**Supplementary Figure 16. Morphology of descending neurons innervating the lateral accessory lobe (LAL) in *Drosophila*.** (**a**) VGlut-F-200356, a descending neuron with smooth processes in the lateral accessory lobe (LAL), posterior slope (PS), and varicose processes in PS and gnathal ganglion (GNG) of the ipsilateral hemisphere. The axon descends through the ipsilateral neck connective. Confocal image with tissue signal (a1) and frontal and dorsal views of reconstructed morphology are shown (a2). (**b**) VGlut-F-500726, a descending neuron with smooth processes in the LAL and PS of the ipsilateral hemisphere and varicose processes in the PS and GNG of the contralateral hemisphere. The axon descends through the contralateral neck connective. Confocal image with tissue signal (b1) and frontal and dorsal views of reconstructed morphology are shown (b2). Data were obtained from the FlyCircuit Database (<http://www.flycircuit.tw/>).

**Supplementary Table 1. Morphological characteristics of DNs in the study.**

| cell # *1 | name | cell body *2 | descending side *3 | midline cross *4 | smooth process | varicose process |
| --- | --- | --- | --- | --- | --- | --- |
| 1 | Group-IA | 1 | C | Y | LAL, PS | LAL, PS, GNG |
| 2 | Group-IA | 1 | C | Y | LAL, PS | LAL, PS, GNG |
| 3 | Group-IA | 1 | C | Y | LAL, PS | LAL, PS, GNG |
| 4 | Group-IB | 1 | C | Y | LAL, PS, GNG | LAL, PS, GNG |
| 5 | Group-IB | 1 | C | Y | LAL, PS, GNG | LAL, PS, GNG |
| 6 | Group-IB | 1 | C | Y | LAL, PS, GNG | LAL, PS, GNG |
| 7 | Group-IB | 1 | C | Y | LAL, PS, GNG | LAL, PS, GNG |
| 8 | Group-IC | 1 | C | Y | LAL, PS | LAL, PS, GNG |
| 9 | Group-IC | 1 | C | Y | LAL, PS | LAL, PS, GNG |
| 10 | Group-ID | 1 | I | Y | PS, IB, PLP | GNG |
| 11 | Group-IE | 1 | I | N | Lo, LAL, PS, PLP | PS, GNG |
| 12 | Group-IIA | 2 | I | N | LAL, PS | GNG |
| 13 | Group-IIA | 2 | I | N | LAL, PS | GNG |
| 14 | Group-IIA | 2 | I | N | LAL, PS | GNG |
| 15 | Group-IIA | 2 | I | N | LAL, PS | GNG |
| 16 | Group-IIA | 2 | I | N | LAL, PS | GNG |
| 17 | Group-IIA | 2 | I | N | LAL, PS | GNG |
| 18 | Group-IIB | 2 | I | N | LAL, PS | GNG |
| 19 | Group-IIB | 2 | I | N | LAL, PS | GNG |
| 20 | Group-IIB | 2 | I | N | LAL, PS | GNG |
| 21 | Group-IIB | 2 | I | N | LAL, PS | GNG |
| 22 | Group-IIC | 2 | I | N | PS, IB | GNG |
| 23 | Group-IIC | 2 | I | N | PS, IB | GNG |
| 24 | Group-IIC | 2 | I | N | PS | GNG |
| 25 | Group-IIC | 2 | I | N | PS | GNG |
| 26 | Group-IIC | 2 | I | N | PS | GNG |
| 27 | Group-IIC | 2 | I | N | PS | GNG |
| 28 | Group-IIC | 2 | I | N | PS | GNG |
| 29 | Group-IIC | 2 | I | Y | PS, IB | GNG |
| 30 | Group-IIC | 2 | I | Y | PS, IB | GNG |
| 31 | Group-IID | 2 | I | N | SMP, LAL, PS | GNG |
| 32 | Group-IID | 2 | I | N | SMP, LAL, PS | GNG |
| 33 | Group-IID | 2 | I | N | LAL, PS | GNG |
| 34 | Group-IID | 2 | I | N | SMP, LAL, PS | GNG |
| 35 | Group-IID | 2 | I | N | SMP, LAL, PS | GNG |
| 36 | Group-IID | 2 | I | N | LAL, PS | GNG |
| 37 | Group-IID | 2 | I | N | SMP, LAL, PS | GNG |
| 38 | Group-IID-like | 2 | I | N | LAL, PS | PS, GNG |
| 39 | Group-IIE | 2 | I | N | PS | PS, GNG |
| 40 | Group-IIF | 2 | I | N | LAL, PS, PLP | PS, GNG |
| 41 | Group-III LDN1 | 3 | C | Y | Lo, PLP | PS, GNG |
| 42 | Group-III LDN1 | 3 | C | Y | Lo, PLP | PS, GNG |
| 43 | Group-III LDN1 | 3 | C | Y | Lo, PLP | PS, GNG |
| 44 | Group-III LDN2 | 3 | C | Y | Lo, SMP, LAL, PS, PLP | PS, GNG |
| 45 | Group-III LDN3 | 3 | C | Y | Lo, SMP, LAL, PS, PLP | PS, GNG |
| 46 | Group-III PSDN2 | 3 | C | Y | PS, GNG | GNG |
| 47 | Group-III PSDN2 | 3 | C | Y | PS, GNG | GNG |
| 48 | - | 3 | C | Y | SMP, SLP, ICL | PS, GNG |
| 49 | - | 3 | C | Y | SMP, SLP, ICL, IB | PS, GNG |
| 50 | - | 3 | C | Y | SMP, SLP, ICL, PS, PLP | PS, GNG |
| 51 | Group-III PSDN1 | 3 | I | N | PS, IB, PLP | GNG |
| 52 | Group-III PSDN1 | 3 | I | N | PS, IB, PLP | GNG |
| 53 | Group-III PSDN1 | 3 | I | N | PS, IB, PLP | GNG |
| 54 | - | 3 | I | N | VLP | GNG |
| 55 | - | 3 | I | N | VLP, AOTu | GNG |
| 56 | - | 3 | I | N | SLP | PS, GNG |
| 57 | - | 3 | I | N | PS, ICL, PLP | - |

*1, Name of identified DNs. ‘-’ means that the DN is not named in the present study.

*2, Cell body position of DNs. Cell bodies belonging to the group-I cell cluster (1), group-II cell cluster (2), and group-III on the posterior brain surface (3). Location of the cell bodies is shown in Figure 1.

*3, Side of descending axon. ‘I’ represents ipsilaterally descending neuron, where the descending axon running through the neck connective of the hemisphere which contains the cell body. ‘C’ represents contralaterally descending neuron, where the descending axon running through the neck connective of the hemisphere which does not contain the cell body.

*4, The presence or absence of neurite which cross the midline in the brain. ‘Y’ represents the presence of the neurite crossing midline. ‘N’ represents the absence of such neurite. The innervation is confined within one hemisphere.

**Supplementary Table 2. Abbreviation for anatomical terms.**

| abbreviation | name |
| --- | --- |
| AL | antennal lobe |
| AOTu | anterior optic tubercle |
| Ca | calyx of the mushroom body |
| CBL | central body lower division |
| CBU | central body upper division |
| DN | descending neuron |
| ES | esophagus |
| GNG | gnathal ganglion |
| IB | inferior bridge |
| ICL | inferior clamp |
| LAL | lateral accessory lobe |
| LALC | lateral accessory lobe commissure |
| l-ALT | lateral antennal-lobe tract |
| Lo | lobula |
| LY | Lucifer Yellow |
| m-ALT | medial antennal-lobe tract |
| MB | mushroom body |
| Pe | pedunclus of the mushroom body |
| PLP | posterior lateral protocerebrum |
| PS | posterior slope |
| SLP | superior lateral protocerebrum |
| SMP | superior medial protocerebrum |
| VLP | ventral lateral protocerebrum |
| VPC | ventral protocerebrum |
| ΔILPC | delta area of the inferior lateral protocerebrum |

**Supplementary Video 1: Z stack images of confocal microscopy data of backfill labeling.**

An example result of backfill staining with Lucifer Yellow from the neck connective on both sides. Frontal view of confocal stacks is shown. Images were acquired with 10× objective. Total 134 successive focal planes (1023 µm × 1023 µm) were taken along vertical axis at 2.4 µm interval. Same to the sample shown in Figure 2.
